# Inflammation subsequent to mild iron excess differentially alters regional brain iron metabolism, oxidation and neuroinflammation status in mice

**DOI:** 10.3389/fnagi.2024.1393351

**Published:** 2024-05-21

**Authors:** Azhaar Ahmad Ashraf, Manal Aljuhani, Chantal J. Hubens, Jérôme Jeandriens, Harold G. Parkes, Kalotina Geraki, Ayesha Mahmood, Amy H. Herlihy, Po-Wah So

**Affiliations:** ^1^Department of Neuroimaging, Institute of Psychiatry, Psychology and Neuroscience, King’s College London, London, United Kingdom; ^2^Department of Human Biology and Toxicology, Faculty of Medicine, University of Mons, Mons, Belgium; ^3^Diamond Light Source, Harwell Science and Innovation Campus, Didcot, United Kingdom; ^4^Perspectum Diagnostics Ltd., Oxford, United Kingdom

**Keywords:** iron metabolism, inflammation, lipid peroxidation, cortex, hippocampus, aging, System X_c_^−^, hyper-ramified microglia

## Abstract

Iron dyshomeostasis and neuroinflammation, characteristic features of the aged brain, and exacerbated in neurodegenerative disease, may induce oxidative stress-mediated neurodegeneration. In this study, the effects of potential priming with mild systemic iron injections on subsequent lipopolysaccharide (LPS)-induced inflammation in adult C57Bl/6J mice were examined. After cognitive testing, regional brain tissues were dissected for iron (metal) measurements by total reflection X-ray fluorescence and synchrotron radiation X-Ray fluorescence-based elemental mapping; and iron regulatory, ferroptosis-related, and glia-specific protein analysis, and lipid peroxidation by western blotting. Microglial morphology and astrogliosis were assessed by immunohistochemistry. Iron only treatment enhanced cognitive performance on the novel object location task compared with iron priming and subsequent LPS-induced inflammation. LPS-induced inflammation, with or without iron treatment, attenuated hippocampal heme oxygenase-1 and augmented 4-hydroxynonenal levels. Conversely, in the cortex, elevated ferritin light chain and xCT (light chain of System X_c_^−^) were observed in response to LPS-induced inflammation, without and with iron-priming. Increased microglial branch/process lengths and astrocyte immunoreactivity were also increased by combined iron and LPS in both the hippocampus and cortex. Here, we demonstrate iron priming and subsequent LPS-induced inflammation led to iron dyshomeostasis, compromised antioxidant function, increased lipid peroxidation and altered neuroinflammatory state in a brain region-dependent manner.

## Introduction

Iron is essential for crucial reactions in brain cells, including neurotransmitter, adenosine triphosphate (ATP), and myelin syntheses. If unregulated, excess iron enhances reactive oxygen species (ROS) production and oxidative stress, thus cellular iron levels are under strict homeostatic control. Iron import proteins that regulate cellular iron entry include the transferrin receptor (TfR) and divalent metal transporter 1 (DMT1) (Ashraf et al., 2018). Excess cellular iron is either sequestered by ferritin in a non-toxic yet bioavailable form or exported out of the cell via ferroportin aided by a ferroxidase, ceruloplasmin. In aging and neurodegenerative diseases, the iron homeostatic cascade appears to be disrupted (Ward et al., 2014). Ferroptosis, a recently discovered form of iron-induced cell death (Dixon et al., 2012), is thought to be a significant contributor to neurodegeneration, including in Alzheimer’s disease (AD) (Ashraf and So, 2020; Ashraf et al., 2020).

Previously, we established that iron dyshomeostasis and neuroinflammation are two facets of brain aging in C57Bl/6J mice (Walker et al., 2016; Ashraf et al., 2019a). Iron is known to stimulate microglia via NFκB activation and increase production of pro-inflammatory cytokines (Saleppico et al., 1996). Inflammation can induce iron dyshomeostasis through altering ferritin expression and other players implicated in iron homeostasis (Zhang et al., 2015; Ashraf et al., 2018; Ward et al., 2022). We propose the combination of increasing iron dysregulation and chronic neuroinflammation with aging contributes to advanced age being the major risk factor in neurodegenerative disease. However, the effect of peripheral administration of iron and/or lipopolysaccharide (LPS)-induced inflammation on the young adult mouse brain remains to be understood.

In this study, we initially administered (mild) iron injections into 8-week-old mice. From 8-weeks of age, iron has been shown to enter the mouse brain, albeit relatively slowly and accumulate as brain iron export is minimal (Holmes-Hampton et al., 2012). At this age, the mouse brain has not had the opportunity to accrete iron, or exhibit chronic neuroinflammation, as would be observed in older mice (Walker et al., 2016; Ashraf et al., 2019a). Contrary to previous iron dietary supplementation studies (Sobotka et al., 1996; Malecki et al., 2002), we chose to systemically administer relatively lower iron doses to represent the insidious brain accumulation of iron that is more representative of aging, similar to other previous studies (Maaroufi et al., 2009, 2014). Supplementation of iron via the diet has been as high as 20,000 parts of million (ppm) of carbonyl iron (Sobotka et al., 1996) which approximates to ingestion of 0.86–1.43 mmol iron per day as compared to approximately 0.0002 mmol iron per day as in our and other studies (Maaroufi et al., 2009, 2014). Two weeks after iron treatment, mice underwent a mild LPS dosing regime known to modify brain immunological responses (Ifuku et al., 2012). We induced an inflammatory insult sometime after the systemic iron injections to allow/mimic the accretion of brain iron and subsequent chronic neuroinflammation as seen with aging.

We determined whether proteins involved in iron homeostasis and antioxidation, and lipid peroxidation protein adduct levels were modulated by systemic inflammation/neuroinflammation subsequent to previous acute mild iron doses. Furthermore, we assessed whether iron priming prior to inflammation/neuroinflammation modulates subsequent glial morphology and spatial learning ability. We focused on the hippocampus, cortex and basal ganglia regions, but in particular, the hippocampus and cortex as these regions play major roles in cognition, which is impaired in normal aging and neurodegenerative diseases such as AD (Devanand et al., 2012; Fjell et al., 2014). We also investigated possible changes in the basal ganglia as these brain regions accrete iron to a much greater extent than other brain regions with aging (Hallgren and Sourander, 1958; Ward et al., 2014) and are affected in neurodegenerative diseases (Guan et al., 2022). Prior to brain isolation for metal, western blot and immunohistochemical analyses, mice underwent spatial learning behavioral testing. Total reflection X-ray fluorescence (TXRF) and synchrotron radiation X-ray fluorescence (SRXRF) mapping were used for bulk and spatial iron (metals) analyses, respectively. Ferroptosis-related—iron regulatory and antioxidant proteins, glia-specific proteins, and lipid peroxidation protein adducts were assessed by western blotting. Ionized calcium-binding adaptor molecule 1 (Iba1) and glial fibrillary acidic protein (GFAP) immunohistochemistry, was used to assess microglial morphology and astrogliosis, respectively. Microglia and astroglia are key players in neuroinflammation, and microglial and astroglial measurements were used as putative indicators of neuroinflammation. We hypothesized that iron priming and subsequent neuroinflammation in a normal young mouse model would exhibit protein changes reminiscent of ferroptosis (iron accumulation/dyshomeostasis, impaired antioxidation, lipid peroxidation) in the brain to a greater extent than by systemic iron injections or inflammation alone.

## Materials and methods

### Animals and treatment

Young male C57Bl/6J mice (3-weeks of age, *n* = 56) were obtained from Envigo (Huntingdon, United Kingdom), housed in pairs on arrival, and acclimatized before experimentation. All experimental procedures were approved by the local ethical review panel of King’s College London in accordance with the U.K. Home Office Animals Scientific Procedures Act 1986.

After acclimatization, at 8-weeks of age, mice were injected intraperitoneally with saline (*n* = 27) or 3 mg/kg ferrous sulphate heptahydrate (423731000, Fisher Scientific, Leicestershire, United Kingdom; *n* = 29) daily for 5 days (Maaroufi et al., 2009). Saline- or iron-treated (*n* = 5/group) mice were euthanized two days after the final injection and brains isolated (see below) to study the acute effects of mild iron treatment on the brain (Cohort 1). Similarly, another cohort (saline, *n* = 7; iron-treated, *n* = 8) were killed at three weeks post-dose (aged 12 weeks) and brains isolated (see below) to determine the potential longer term/chronic effect of a single iron treatment (Cohort 2). The remaining saline- or iron-treated mice were subdivided and received either saline (10 mg/kg) or 0.25 mg/kg LPS (L2880, Sigma, Dorset, United Kingdom) intraperitoneally daily for a week, yielding four final groups (Cohort 3): saline only (control group, *n* = 7), iron only (*n* = 8), LPS only (*n* = 8) and iron + LPS group (*n* = 8). Two weeks after the final injection, mice (aged 15-weeks) underwent spatial learning behavioral (cognitive) assessment (see behavior testing) and then brains isolated (see below).

**Figure 1:**
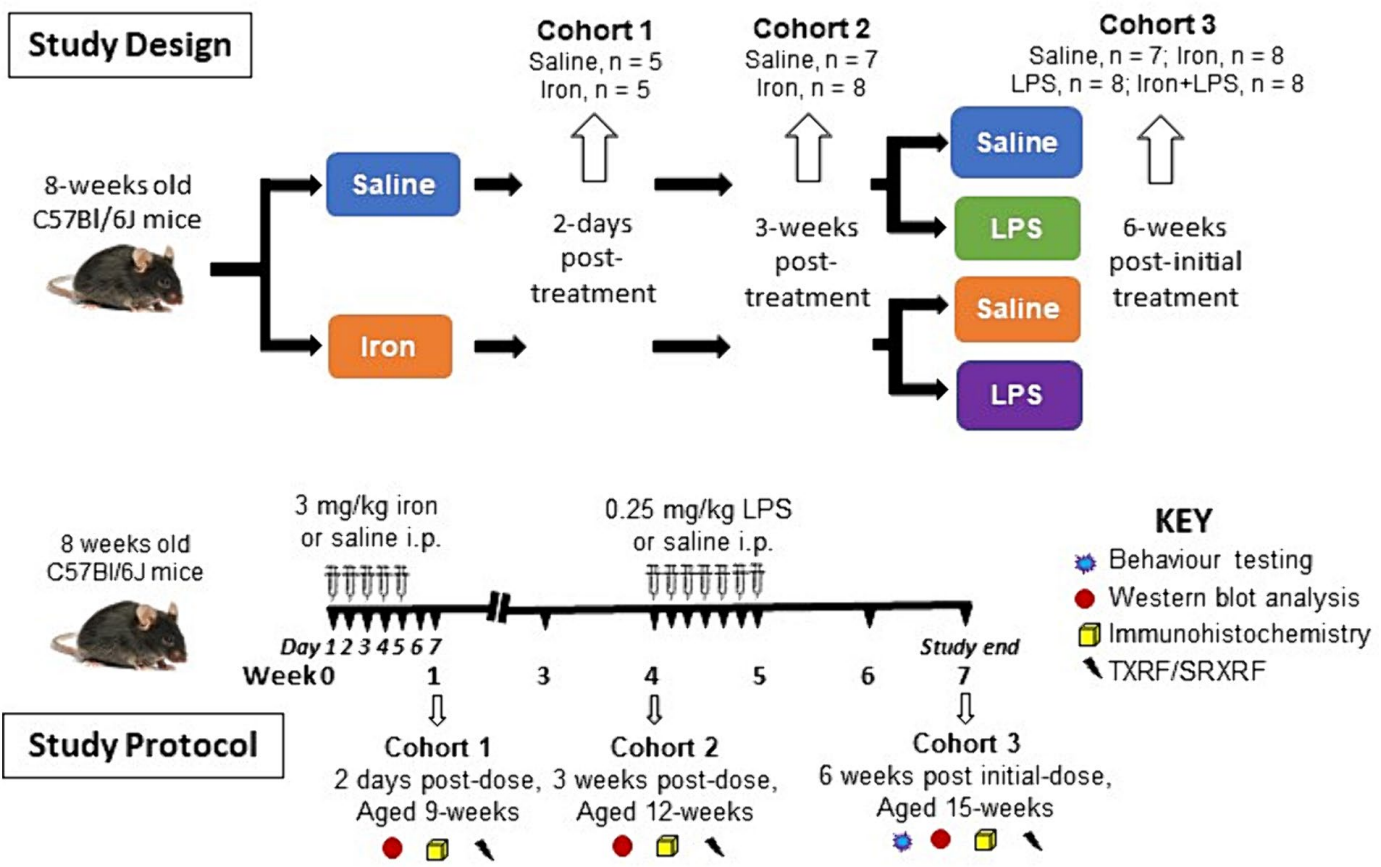
Overview of the study design and study protocol. i.p., intraperitoneally; LPS, lipopolysaccharide; SRXRF, synchrotron-radiation X-ray fluorescence; TXRF, total reflection X-ray fluorescence.

Brains were harvested after exsanguination by transcardial perfusion with phosphate-buffered saline (PBS) under terminal isoflurane-oxygen anesthesia. Hippocampus, cortex, striatum, and substantia nigra were dissected from the right-brain hemisphere and snap-frozen in liquid nitrogen and stored at −80°C for western blotting and TXRF (see below). (Note, the substantia nigra was not dissected out for Cohort 1.) The remaining left-brain hemisphere was fixed in 4% paraformaldehyde in PBS for 48 h before storage in PBS-0.025% sodium azide at 4°C for immunohistochemistry and SRXRF (see below). An overview of the study design and protocol is shown in Figure 1.

### Behavioral (cognitive) testing

Novel object location (NOL) testing was used to evaluate learning and memory in mice, as described previously (Lueptow, 2017). Briefly, testing was performed in an opaque arena (40 cm × 40 cm, and 50 cm high walls), and exposed to light intensity of 50 lux and ambient noise, ~65 dB (supplied by a radio). All behavior trials were recorded using EthoVision XT software (Noldus information technology, VA, United States). Equipment between trials were wiped clean and treated with 0.1% acetic acid to eliminate residual odors.

Mice were placed into the arena and allowed to explore two identical objects for 10 min (training trial) and then returned to their home cage. An hour later, they were re-introduced into the arena after one of the objects had been moved to a novel location and allowed to explore again for 10 min (test trial). The total time each animal spends exploring each object, characterized by active sniffing, or rearing against the object with the nose directed towards the object within 2–3 cm of the object, was recorded. The center-point of the mice defined its position for analysis. We calculated the discrimination index for NOL using Eq. 1:


(1) $$\mathrm{Discrimination}\;\mathrm{index}\;\left(DI\right)=\frac{T_{\mathrm{novel}}-T_{\mathrm{familiar}}}{T_{\mathrm{novel}}+T_{\mathrm{familiar}}}$$

where *T*_novel_ and *T*_familiar_ are times spent exploring a novel and familiar location, respectively.

Mice with preserved spatial memory and curiosity will spend more time exploring the object placed in a novel location compared with the familiar location (Lueptow, 2017). A higher discriminatory index (DI) score implies a preference for the novel location, suggesting intact spatial memory and recognition of the change of object location.

### Western blotting

For western blotting, lysates of hippocampus, cortex, and striatum were prepared as before (Ashraf et al., 2020), and stored at −80°C until further analysis. Brain lysates (40 μg protein) were mixed with Laemmli buffer (S3401; Sigma, Poole, United Kingdom) and heated (95°C, 5 min). Proteins were separated on NovexTM tris-glycine 4–20% gradient gels (XP04205BOX, Thermo Fisher; 150 V, 90 min), and then transferred onto a 0.45 μm nitrocellulose membrane (GE10600002, GE Healthcare, Amersham, United Kingdom) in tris-glycine buffer supplemented with 20% methanol (80 V, 1 h). Following incubation in a blocking solution (1 h, ambient temperature), membranes were incubated with primary antibodies (overnight, 4°C; Supplementary Table S1) for iron-homeostatic proteins: ferritin-light chain (FTL), ferritin-heavy chain (FTH), transferrin-receptor (TfR), DMT1, iron responsive element binding protein 2 (IRP2), ceruloplasmin, ferroportin, heme-oxygenase-1 (HO-1); for immune cells/inflammation, Iba1, GFAP, NADPH oxidase 2 (NOX2) and triggering receptor expressed on myeloid cells 2 (TREM2); and for ferroptosis, acyl-CoA synthetase long-chain family member 4 (ACSL4), light chain subunit of System X_c_^−^ (xCT), glutathione peroxidase 4 (GPX4) and the lipid-peroxidation marker 4-hydroxynonenal (4-HNE).

After incubation with primary antibodies, protein bands were visualized as described before (Ashraf et al., 2020) with horse-radish peroxidase (HRP)-conjugated secondary antibodies and imaged (BioRad ChemiDoc MP system). For protein quantification, membranes were stripped using Restore Western blot stripping buffer (20159; Thermo Fisher) and re-probed with HRP-conjugated β-actin. Band signal intensities were quantified by densitometry (ImageJ, NIH), and normalized to β-actin.

### Total reflection X-ray fluorescence

Brain lysates (hippocampus, cortex and striatum) prepared as detailed above were thawed and diluted 1:5 v/v with Milli-Q distilled water and underwent TXRF as described previously (Ashraf et al., 2020). Briefly, following dilution with polyvinyl alcohol (2 μL, 0.3 g/L water; 843871, Merck, Gillingham, United Kingdom), gallium solution (10 μL, 440 μg/L; TraceCERT^®^ gallium standard for inductive coupled plasma-mass spectrometry; 16639, Merck) was added (final gallium concentration of 200 μg/L). Resultant duplicate samples were placed on acrylic sample carriers for TXRF (PICOFOX, Bruker Nano GmbH, Germany) (Ashraf et al., 2019b). Elemental concentrations of iron, copper and zinc were calculated by reference to the internal gallium standard and normalized to protein concentrations (mg/kg protein) (Ashraf et al., 2020).

### Synchrotron radiation X-ray fluorescence

Fixed brain samples were cryoprotected in 30% sucrose in PBS for 48 h and frozen at −20°C before cryosectioning. Cryosections (30 μm thick) were mounted onto 4 μm thick Ultralene film (Spex Sample-Prep, NJ, United States) secured to a customized holder and kept at room temperature prior to SRXRF.

Brain cryosections containing the hippocampus, cortex, striatum, and substantia nigra (*n* = 4/group in Cohort 2 and *n* = 7–8/group in Cohort 3) underwent SRXRF at the Diamond Light Source synchrotron radiation facility (microfocus beamline I18; Didcot, United Kingdom), as described previously (Ashraf et al., 2019a). Briefly, cryosections were mounted at a 45° angle with respect to the incoming X-ray beam and the detector, and scanned raster fashion with a beam of 100 μm diameter (resolution) and 11 keV energy. Full energy dispersive spectra were collected for each beam position, deconvolved and areas of the characteristic peaks of iron, zinc, and copper evaluated using PyMca (Solé et al., 2007) to produce elemental metal maps. For quantification, photon flux was estimated by measurement of a reference metal film (AXO, Dresden, GmbH). Regions of interest (ROIs) were placed on the elemental metal maps to obtain metal concentrations (mg/kg) in different brain regions (Supplementary Figure S1).

### Immunohistochemistry

Immunohistochemistry was performed in the hippocampus, cortex, striatum and substantia nigra from all mice of all cohorts. Brain cryosections were prepared as detailed for SRXRF, albeit sectioned into wells of 96-well plates containing PBS-0.025% sodium azide (see above) and stored at 4°C prior to analysis. For immunohistochemistry, cryosections were treated with 1% hydrogen peroxide in PBS-0.2% TritonX-100, washed with PBS-0.2% Triton X-100 (2 × 5 min), and blocked in 10% BSA supplemented with PBS-0.2% Triton X-100. Antibodies for Iba1 (microglia, SAB2500042; Sigma) and GFAP (astrocytes; Z0334; DAKO) were incubated overnight (5% BSA in PBS-Triton X-100, 4°C), washed, and then incubated with a secondary biotinylated anti-rabbit antibody (5% BSA in PBS-0.2% Triton X-100, 2 h). Following enhancement with avidin-biotin complex (Vectashield, Vector Laboratories, Burlingame, United States) for 30 min, 3,3′-diaminobenzidine (DAB) was used to develop the staining for 4 min and the reaction was stopped by the addition of distilled water. The sections were then mounted on Superfrost slides (J1800AMNZ; Thermo Fisher Scientific), dehydrated sequentially in 70% ethanol (2 min), 90% ethanol (2 × 2 min), 100% ethanol (2 × 2 min), and xylene before mounting in DPX. Images were acquired with a Leica microscope using a 40× objective connected to a camera using Image-Pro software (National Institutes of Health, Bethesda, Maryland, United States). The stained images were quantified using Fiji/ImageJ software.

Microglial morphology was assessed of Iba1-stained brain tissue section images using the skeletonize plugin for ImageJ (Morrison and Filosa, 2013; Young and Morrison, 2018; Ali et al., 2019). The image of each hemisphere section was skeletonized, and microglial branch endpoints and lengths measured across the image (a cutoff value of 20 for endpoints and lengths was used). The endpoint measure assesses the number of microglial branches or processes, while branch length measures their length. Microglial endpoints and branch lengths were normalized to the microglial soma number to give endpoints/cell and branch length/cell, respectively.

For GFAP-stained brain sections, images were converted to an 8-bit greyscale image, a threshold applied, and then converted to binary format in ImageJ. Signal intensities in an optical field were obtained after applying a thresholding protocol using negative control sections (incubated with secondary antibody only) to identify non-specific background staining. Depending on the brain region analyzed, GFAP-immunoreactivity was analyzed in 2–4 optical fields (Walker et al., 2016).

### Statistical analysis

Data normality was assessed using Q-Q, residual, and homoscedasticity plots: values violating these assumptions were identified as outliers and excluded from the analysis.

A two-tailed student’s *t*-test was performed on proteins and metals to monitor the acute and chronic effects of saline- and iron-treatment (Cohorts 1 and 2). To correct for multiple *t*-test comparisons, we used an adaptive linear set-up procedure to control the false discovery rate (FDR) at 5%. One-way ANOVA followed by Tukey *post hoc* correction was used to identify the effects of iron only, inflammation only, and iron-primed inflammation on behavioral performance; levels of iron regulatory, ferroptosis-related, and glial-specific proteins; and metals (Cohort 3). Adjusted *p*-value ≤0.05 were considered significant.

All statistical analyses were performed using IBM SPSS Statistics 26 and GraphPad Prism 9.

## Results

### Acute (Cohort 1) and chronic (Cohort 2) effects of iron

Changes in protein, glial cell, and metal measurements at 2 days (Cohort 1) or 3 weeks (Cohort 2) post-iron injection did not surpass FDR-correction in the different brain regions (Supplementary Tables S2–S9).

### Effects of iron and/or inflammation treatment (Cohort 3)

**Figure 2:**
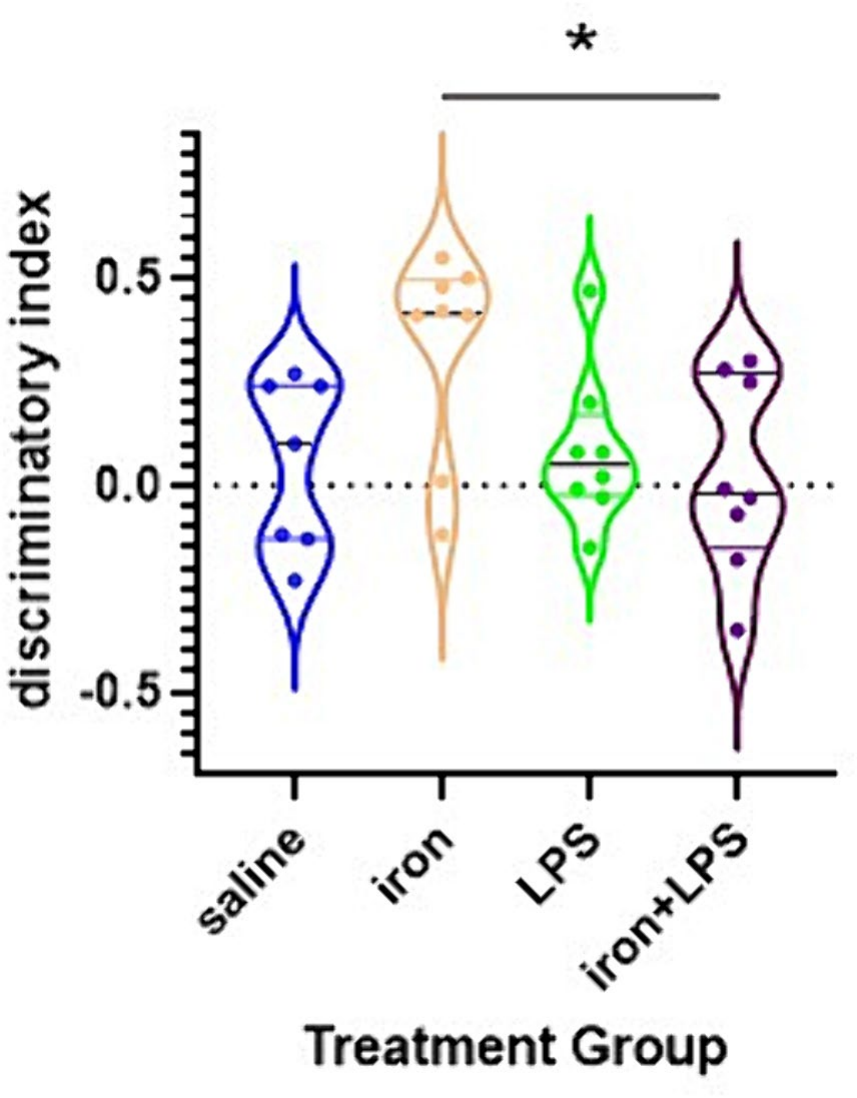
Effects of iron-priming and inflammation on the novel object location (NOL) assessment. *p < 0.05.

The iron only treatment group showed improved NOL performance compared with iron + LPS group (*p* = 0.0440; Figure 2). However, NOL performance was not significantly improved with iron treatment when compared with saline (*p* = 0.0924) or LPS only (*p* = 0.1323) groups.

**Figure 3:**
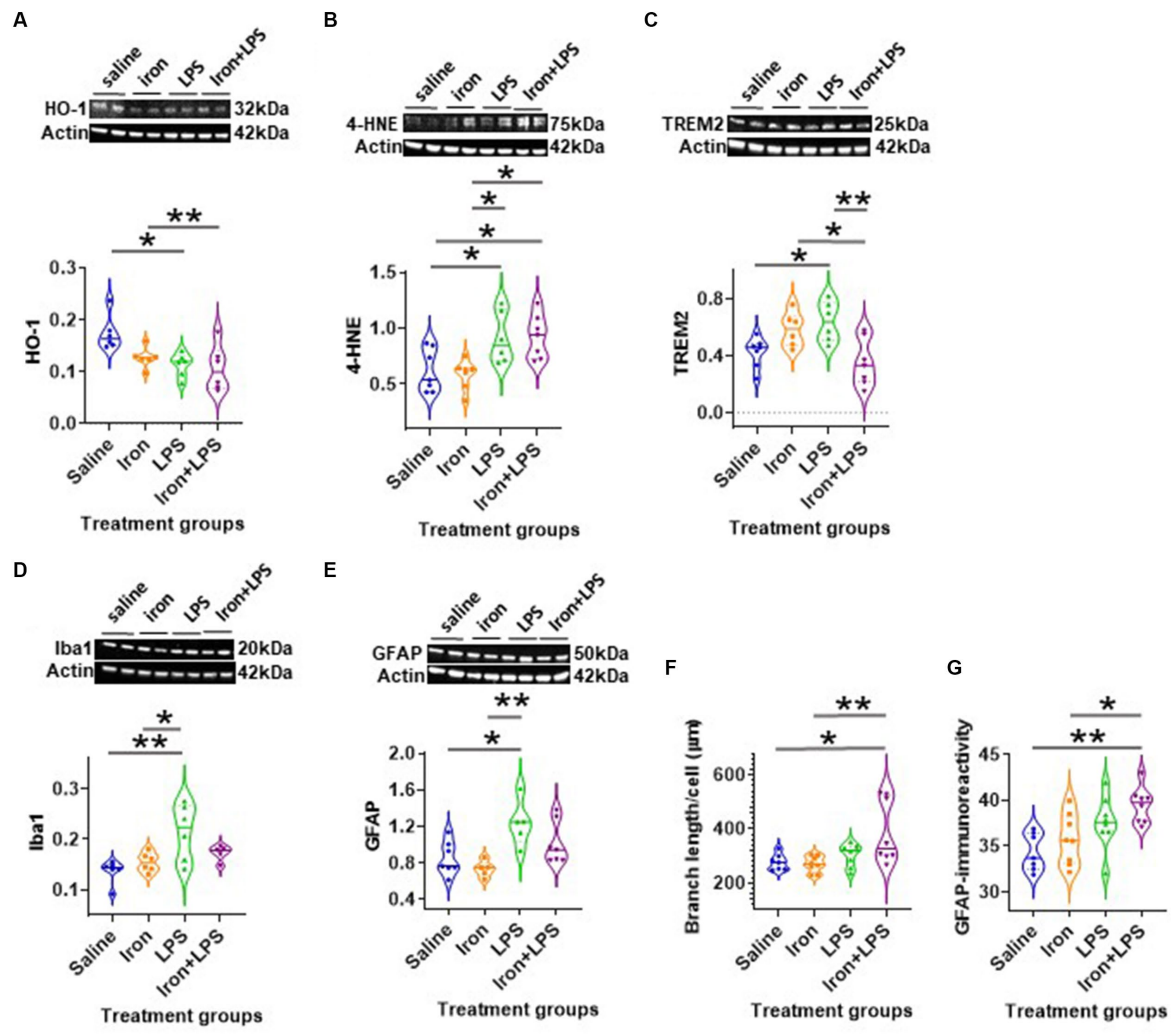
Effect of iron-priming and inflammation in the hippocampus (Cohort 3). Western blot analysis of **(A)** Heme oxygenase-1 (HO-1), **(B)** 4-hydroxynonenal (4-HNE), **(C)** triggering receptor expressed on myeloid cells 2 (TREM2), **(D)** ionized calcium-binding adaptor molecule 1 (Iba1) and **(E)** glial fibrillary acidic protein (GFAP). Immunohistochemical analysis of **(F)** microglial branch length/cell (assessed by Iba1 staining) and **(G)** GFAP signal intensity (SI, arbitary units, a.u.). (Proteins used in western blot were normalised against actin). *p < 0.05 and **p < 0.01. LPS, lipopolysaccharide.

By western blot analysis, LPS-induced inflammation, with (*p* = 0.0061) or without iron treatment (*p* = 0.0149) was shown to attenuate hippocampal HO-1 compared with saline (Figure 3A and Supplementary Table S10). LPS only group and iron + LPS group demonstrated augmented 4-HNE levels compared with both saline (*p* = 0.0462, *p* = 0.0294, respectively) and iron only groups (*p* = 0.0220, *p* = 0.0132, respectively; Figure 3B and Supplementary Table S10). Hippocampal TREM2 levels were found to be higher in the LPS group compared to both saline (*p* = 0.0398) and iron + LPS (*p* = 0.0040) groups, while the iron + LPS group had lower TREM2 compared to the iron only group (*p* = 0.0218; Figure 3C). The LPS group showed increased levels of Iba1 compared with the saline (*p* = 0.0035) and iron (*p* = 0.0247) groups (Figure 3D). Similarly, LPS group demonstrated augmented levels of GFAP than saline (*p* = 0.0174) and iron (*p* = 0.0039) groups (Figure 3E). None of the other measured proteins were found to be altered by the various treatments (Supplementary Table S10). Similarly, metals—iron, zinc, and copper, as assessed by bulk and spatial analyses, TXRF and SRXRF, were comparable between groups in Cohort 3.

**Figure 4:**
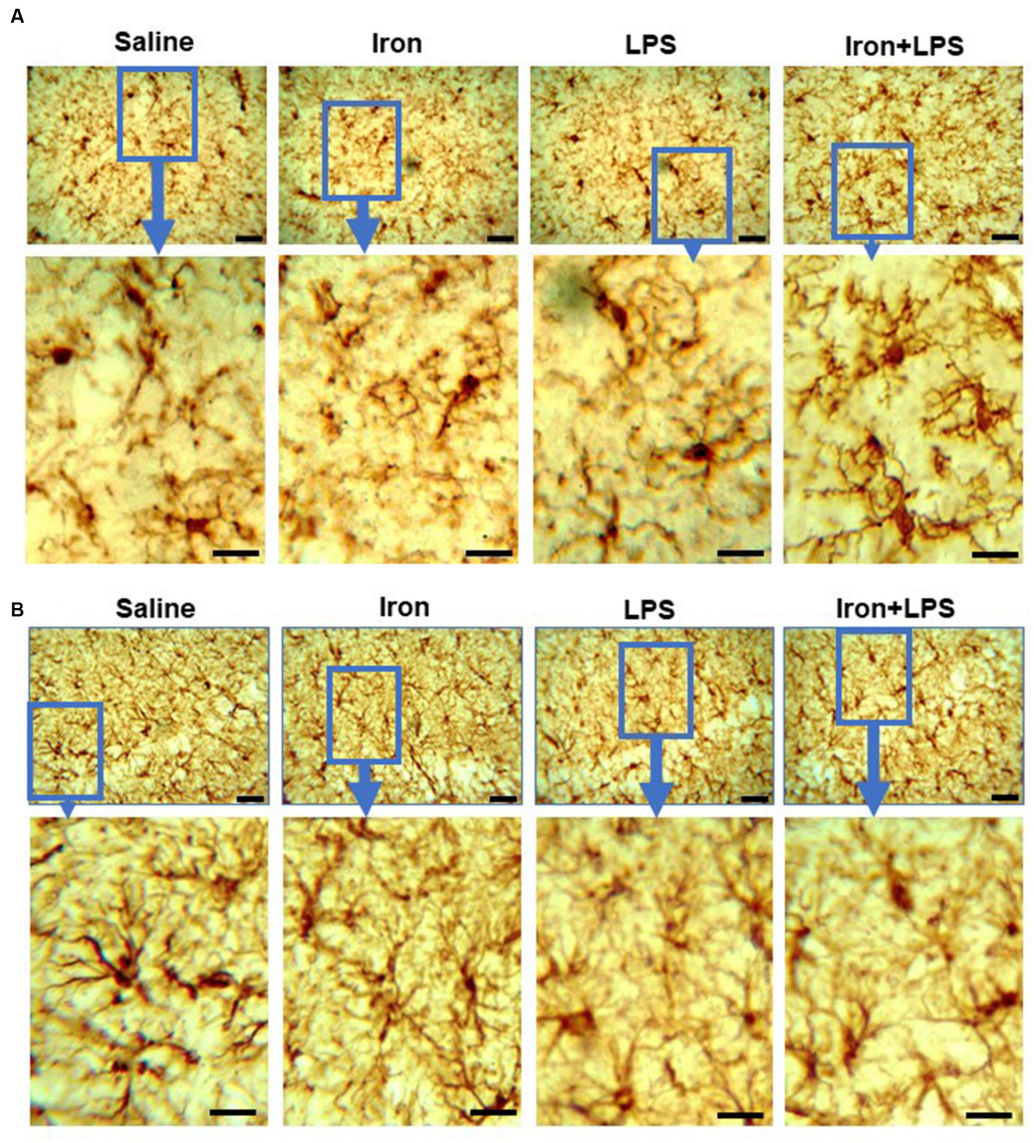
Effect of iron-priming and inflammation on micro- and astro-glial morphology in the hippocampus (Cohort 3). **(A)** ionized calcium-binding adaptor molecule 1 and **(B)** glial fibrillary acidic protein immunochemistry. Micrographs were acquired at 40× magnification. Scale bar represents 25 μm (top panels) and 12.5 μm (bottom panels, magnified images). LPS, lipopolysaccharide.

Analysis of Iba-1 stained immunohistochemical stained images revealed longer hippocampal microglial branch length/cell in the iron + LPS group compared with saline (*p* = 0.0158) or iron (*p* = 0.0071) groups (Figures 3F, 4A). Similarly, augmented GFAP-immunoreactivity was observed in the iron + LPS group compared with either saline (*p* = 0.0022) or iron (*p* = 0.0251) groups (Figures 3G, 4B). However, microglial endpoints/cell were similar between groups (*p* = 0.25; Supplementary Table S10).

**Figure 5:**
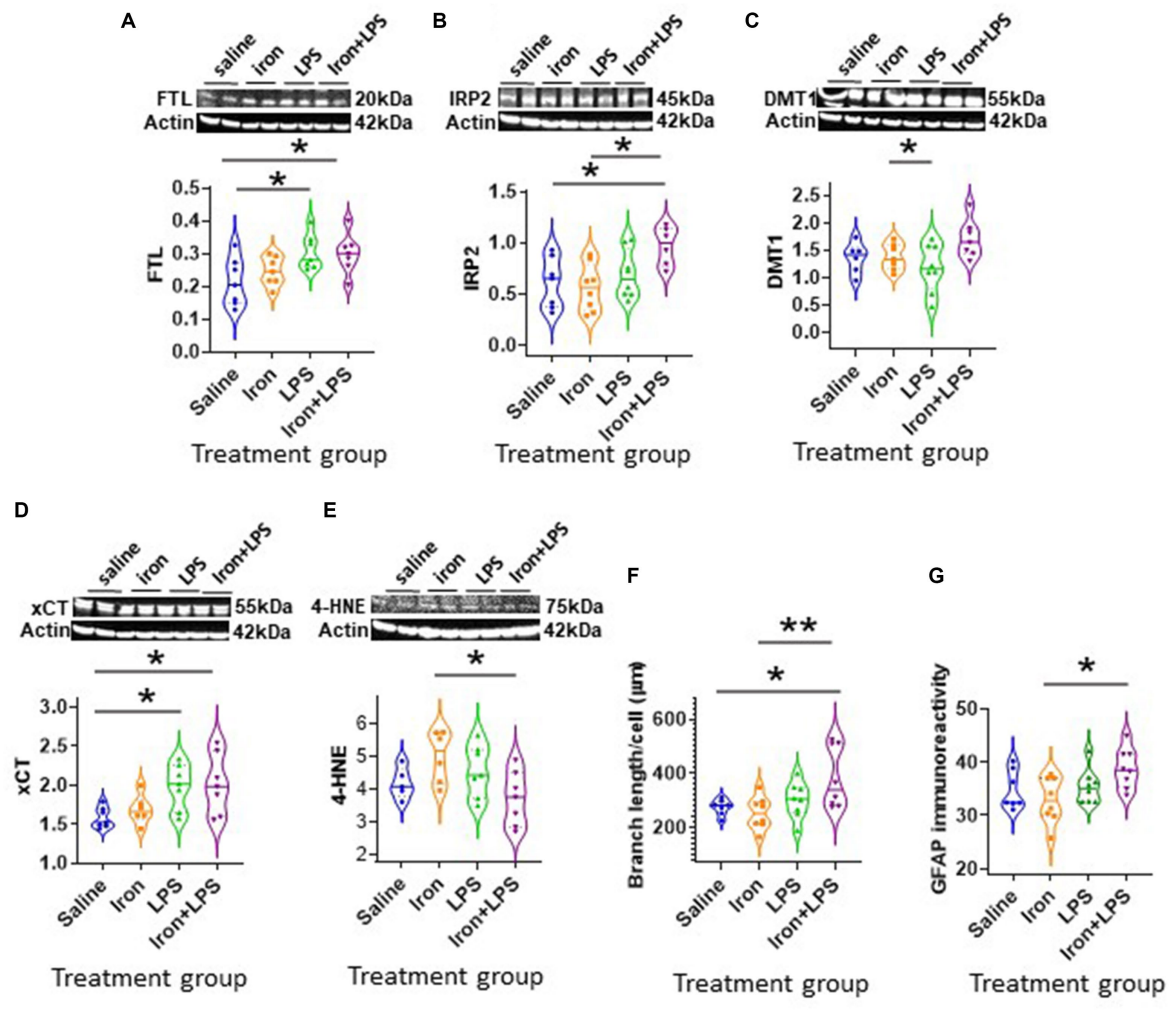
Effect of iron-priming and inflammation in the cortex (Cohort 3). Western blot analysis of **(A)** ferritin light-chain (FTL), **(B)** iron regulatory protein 2 (IRP2), **(C)** divalent metal transporter 1 (DMT1), **(D)** light-chain subunit of system X_c_^−^ (xCT), **(E)** 4-hydroxynonenal (4-HNE). Immunohistochemical analysis of **(F)** microglial branch length/cell (assessed from ionized calcium binding adaptor molecule 1-immunohistochemistry) and **(G)** glial fibrillary acidic protein (GFAP) signal intensity (SI, measured in arbitrary units, a.u.). (Proteins measured by western blot analysis were normalized against actin.) Significant results were * and ** for *p* < 0.05 and 0.01, respectively. LPS, lipopolysaccharide.

The cortex exhibited different changes compared with the hippocampus. Cortical FTL was significantly increased in response to LPS-induced inflammation, with *p* = 0.0498 and *p* = 0.0343, when comparing with and without iron priming to the saline group, respectively (Figure 5A). Meanwhile, IRP2 was higher in the iron + LPS group compared to saline (*p* = 0.0382) and iron (*p* = 0.0131; Figure 5B, Supplementary Table S11). Combinatorial iron + LPS treatment increased cortical DMT1 compared to LPS only treatment (*p* = 0.0299, Figure 5C). Increased xCT in the cortex was observed in response to LPS-induced inflammation, without and with iron-priming compared to saline (*p* = 0.0464 and *p* = 0.0306, respectively; Figure 5D). Surprisingly, the lipid peroxidation marker, 4-HNE, was attenuated in the iron + LPS group relative to iron (*p* = 0.0274, Figure 5E). Levels of other proteins or metals measured were similar irrespective of treatments (Supplementary Table S11).

**Figure 6:**
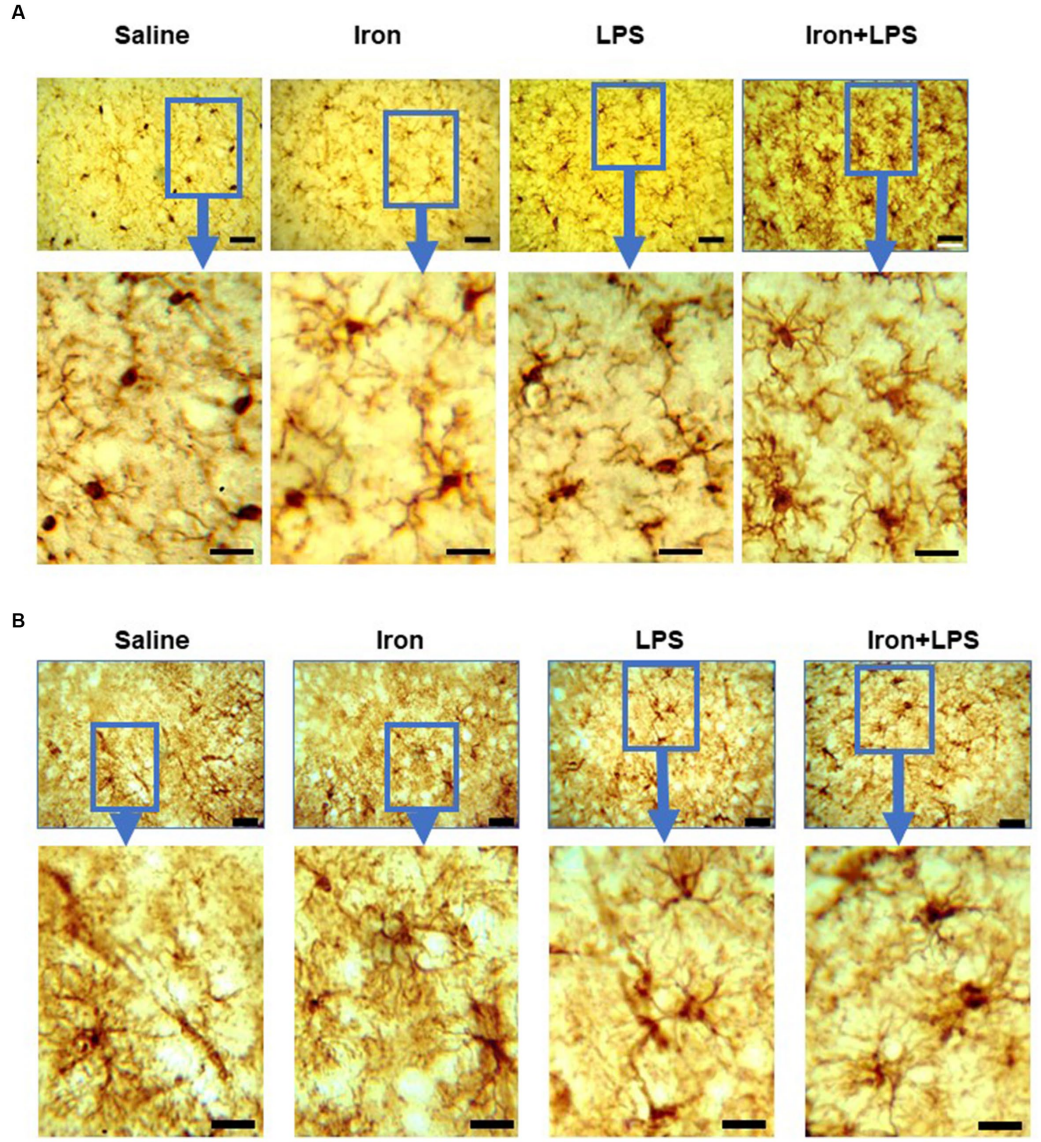
Effect of iron-priming and inflammation on micro- and astro-glial morphology in the cortex (Cohort 3). **(A)** ionized calcium-binding adaptor molecule 1 and **(B)** glial fibrillary acidic protein immunochemistry. Micrographs were acquired at 40× magnification. Scale bar represents 25 μm (top panels) and 12.5 μm (bottom panels, magnified images). LPS, lipopolysaccharide.

By Iba1-immunohistochemistry, cortical microglial endpoints/cell were found to be comparable between groups (*p* = 0.35, Supplementary Table S11). However, longer microglial branch length/cell was observed in the iron + LPS group compared to saline (*p* = 0.0422) and iron only (*p* = 0.0081) groups (Figure 5F, 6A). Further, cortical GFAP-immunoreactivity was augmented in the iron+LPS group compared to the iron only group (*p* = 0.0187; Figures 5G, 6B).

**Figure 7:**
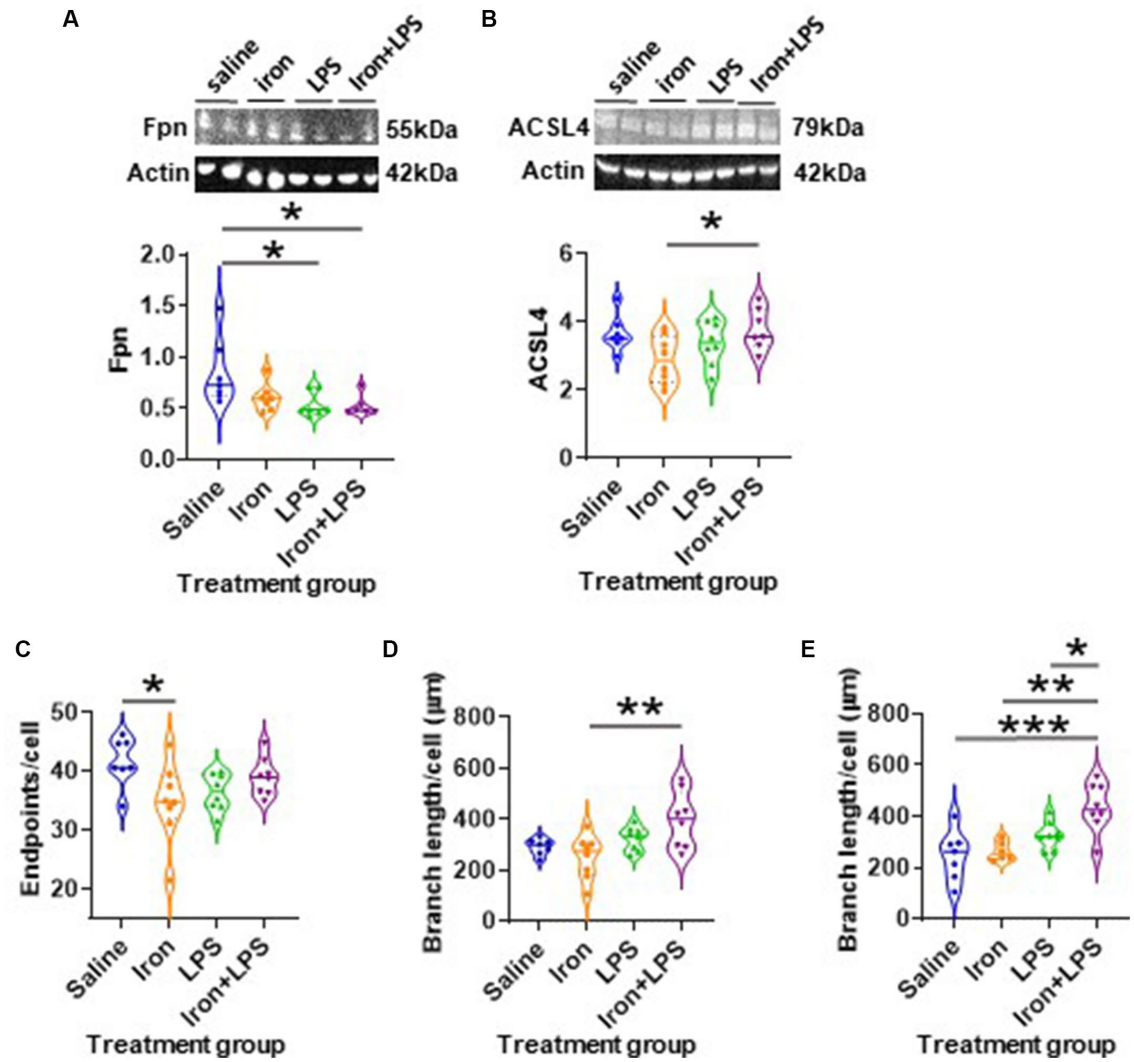
The effects of iron-priming and inflammation in the striatum and substantia nigra (Cohort 3). Western blot analysis of **(A)** ferroportin (Fpn) and **(B)** acyl-CoA synthetase long-chain family member 4 (ACSL4). Ionized calcium binding adaptor molecular 1-immunohistochemical analysis of **(C)** microglial endpoints/cell and **(D)** branch length/cell in the striatum and **(E)** microglial branch length/cell in the substantia nigra. (Proteins measured by western blot analysis were normalized against actin.) Significant results were *, ** and *** for *p* < 0.05, 0.01 and 0.001, respectively. LPS, lipopolysaccharide.

**Figure 8:**
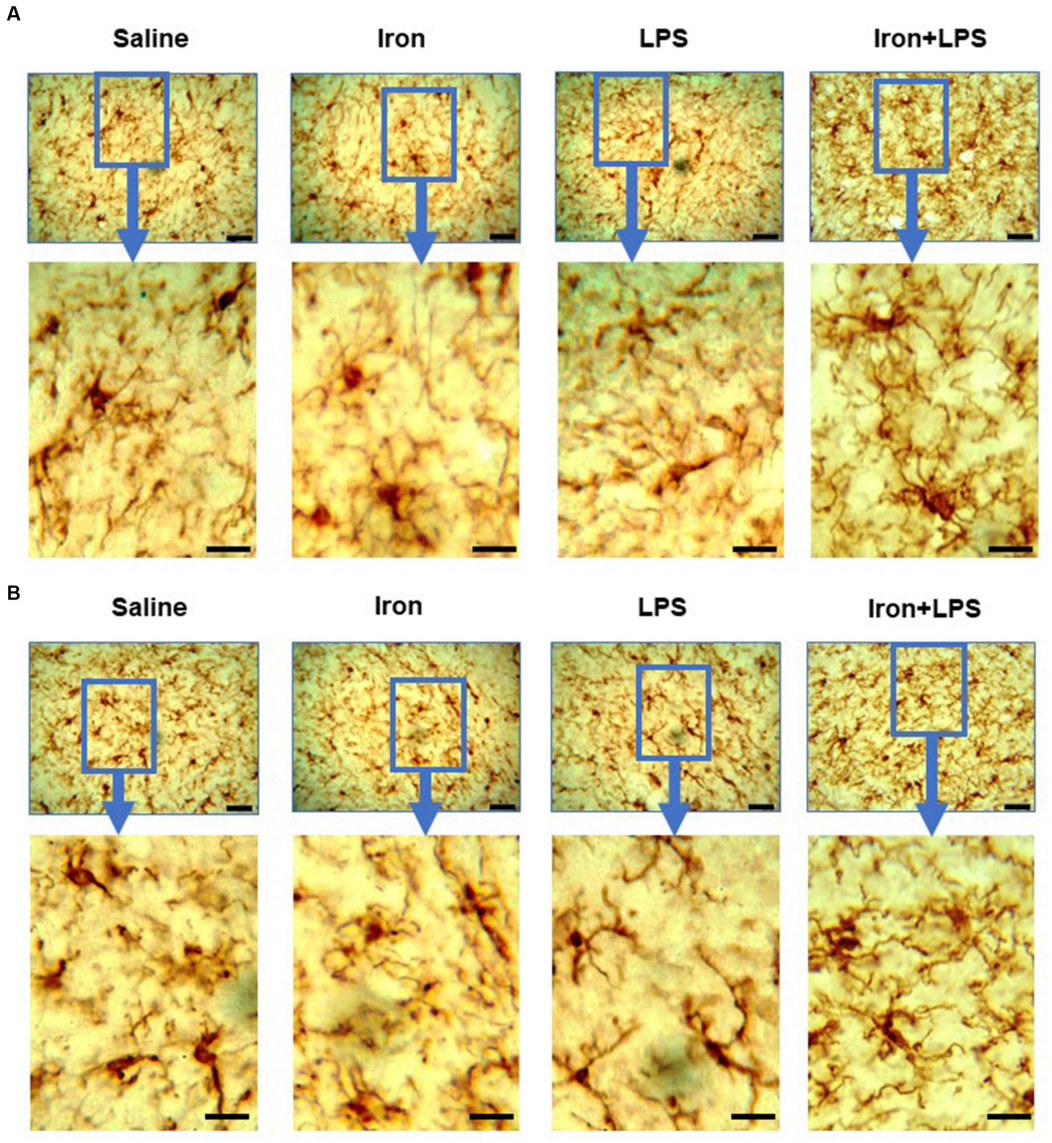
Effect of iron-priming and inflammation in the striatum (Cohort 3) on **(A)** ionized calcium-binding adaptor molecule 1 and **(B)** glial fibrillary acidic protein immunochemistry. Micrographs were acquired at 40× magnification. Scale bar represents 25 μm (top panels) and 12.5 μm (bottom panels, magnified images). LPS, lipopolysaccharide.

The response of the striatum to iron and/or LPS treatment was different from that in both the hippocampus and cortex. Striatal ferroportin levels were decreased in response to LPS-induced inflammation without (*p* = 0.0315) and with iron priming (*p* = 0.0304) compared to saline treatment, respectively (Figure 7A). Levels of ACSL4 were increased in the iron + LPS group relative to the iron only group (*p* = 0.0474; Figure 7B and Supplementary Table S12). Striatal microglial endpoints/cell were decreased by iron-treatment compared to the saline group (*p* = 0.0315, Figure 7C). Conversely, microglial branch length/cell were longer in the iron+LPS treated mice compared to those only treated with iron (*p* = 0.0038; Figures 7D, 8A and Supplementary Table S12).

The substantia nigra also exhibited a regional-specific response to iron and/or LPS. Longer nigral microglial branch lengths were observed in the iron + LPS group compared to saline (*p* = 0.0004), iron (*p* = 0.0018), and LPS groups (*p* = 0.0403, Figures 7E, 8B and Supplementary Table S13). However, the microglial endpoints/cell (*p* = 0.53, Supplementary Table S14) and GFAP-immunoreactivity (*p* = 0.32, Supplementary Table S13) were similar between groups. Likewise, metal levels, including iron, were also similar between groups (Supplementary Table S14).

## Discussion

We demonstrate iron dyshomeostasis, neuroinflammation, inhibition of System X_c_^−^ (cystine/glutamate antiporter), and modulation of lipid peroxidation in young C57Bl/6J mice exposed to peripheral iron and subsequent LPS-induced inflammation, in a brain region-dependent manner.

We reveal neuroprotective effects from a previous short duration of relatively mild systemic iron treatment in young C57Bl/6J mice (Cohort 3), with improved spatial learning on the NOL task, when compared with mice treated with both iron + LPS. We suggest that mild systemic iron injections in mice led to iron being partitioned beneficially towards fulfilling metabolic demands (e.g., iron-dependent brain maturation processes including synaptogenesis) rather than enhancing the labile iron pool and potentially, inducing iron toxicity via lipid peroxidation and oxidative stress. This is consistent with our molecular findings where iron only treatment demonstrated a significant attenuation of hippocampal lipid peroxidation 7-weeks after iron-treatment compared with iron + LPS and LPS only groups. Lipid peroxidation was assessed by measurement of 4-HNE, a reactive aldehyde that can detrimentally alter signaling pathways in aging (Zhang and Forman, 2017).

The brain is particularly susceptible to lipid peroxidation-mediated oxidative stress due to its high oxygen consumption and lipid-rich content. Mice subjected to LPS-induced inflammation, with or without iron-priming, demonstrated augmented lipid peroxidation. This may be attributed to the reduced HO-1 levels observed in LPS-treated mice, irrespective of prior treatment with iron or not. HO-1 is an antioxidant enzyme that catalyzes the detoxification/degradation of heme (from hemoglobin) to ferrous iron (to be sequestered by ferritin), biliverdin/bilirubin (lipid antioxidant), and carbon monoxide (Eskew et al., 1999; Sung et al., 2000; Vanacore et al., 2000; Wu and Hsieh, 2022). HO-1 attenuates the generation of ROS (e.g., superoxide) and lipid peroxidation (Chao et al., 2013), and an integral part of the antioxidant system: HO-1 null mice exhibit increased lipid peroxides (Ishikawa et al., 2012). Diminished HO-1 levels (and enzymatic activity) prevent heme-detoxification and increases heme availability for the generation of ROS, thereby increasing the susceptibility of cells to oxidative stress (Pirota et al., 2016; Righy et al., 2016; Chiabrando et al., 2018).

We showed that LPS only treatment increased hippocampal levels of Iba1 (microglia) and GFAP (astrocytes) compared with saline and iron only treatments. Increased numbers of microglia and astrocytes have been previously demonstrated in response to LPS administration in mice (Ifuku et al., 2012). LPS has been shown to augment pro-inflammatory cytokine secretion from microglia and astrocytes (Godbout et al., 2005; Qin et al., 2007; Henry et al., 2009; Ifuku et al., 2012; Wohleb et al., 2012). LPS likely induces microglia to secrete IL1β which in turn stimulates astrocytic levels/activation (Norris et al., 2005; Sama et al., 2008; Perry et al., 2010).

Mice receiving LPS after iron-priming exhibited longer microglial branch length/cell compared to saline and iron only groups. Little is known about the initiation of microglial hyper-ramification (i.e., branching) and how this phenotype relates to microglial functionality (Hinwood et al., 2013). However, exposure of mice to chronic psychological stress, e.g., depression or post-traumatic stress disorder, in the absence of injury or neurodegeneration, has been shown to increase microglial ramification (unaltered microglial numbers) in various brain regions (Hinwood et al., 2013; Walker et al., 2013; Hellwig et al., 2016; Smith et al., 2019). Anti-depressant treatment which ameliorates depression like-behavior was shown to attenuate microglial hyper-ramification and restore microglial morphology. Moreover, microglia exhibited hyper-ramification in a DNA-repair deficient model of accelerated aging (Ercc1 mutant mice) (Raj et al., 2014). Microglia in these mice displayed an exaggerated response to LPS stimulation with augmented expression of pro-inflammatory cytokines (IL1β, IL6, and TNFα) and generation of ROS. We suggest that a LPS challenge following iron-priming leads to hyper-ramified microglia which may represent a primed microglial state. Primed microglia are not acutely activated but may exhibit differential expression patterns, e.g., increased CD68, and mount an exaggerated inflammatory response to a subsequent immune challenge (Witcher et al., 2015). Notably, acute iron loading in mouse macrophages has been found to potentiate the inflammatory response to a consequent LPS challenge (Hoeft et al., 2017). Moreover, pre-treatment of macrophages with an iron-chelator in normal mice attenuated the LPS-induced inflammatory response (Wang et al., 2009). In primary cultured ventral mesencephalic (VM) neurons, augmented release of IL1β and TNFα was observed in LPS-activated microglia exhibiting iron overload (Wang et al., 2013). Iron treatment increases microglial secretion of proinflammatory cytokines which can augment ROS and lipid peroxidation (Yang et al., 2007, 2013; Hoeft et al., 2017; Yauger et al., 2019). Our study demonstrates that excess iron and subsequent inflammation alters hippocampal microglial morphology (observed as hyper-ramification of processes) alongside increased lipid peroxidation.

Decreased hippocampal TREM2 was observed following LPS treatment, but only with iron priming, suggesting that iron-primed hippocampal microglia may not be able to respond appropriately to a subsequent inflammation stimulus, e.g., attenuated phagocytosis. TREM2 is exclusively expressed by microglia and is known to promote phagocytic function and regulate inflammation (Takahashi et al., 2005, 2007; Hsieh et al., 2009). TREM2 is pivotal in sustaining trophic functions of microglial in the aging brain (Poliani et al., 2015) and microglial TREM2 expression has been to be reduced in aged mice (Hickman et al., 2013). Further, loss of function mutations of TREM2 is associated with Nasu-Hakola disease, an inflammatory degenerative disease of the brain and bone, leading to premature dementia and death (Bianchin et al., 2010). Rare variants of TREM2 have augmented risk of developing late-onset AD (Jonsson et al., 2013).

Hippocampal GFAP-immunoreactivity was also significantly increased in iron-primed mice subjected to a subsequent LPS challenge, which has also been observed in the aging mouse brain (Kohama et al., 1995). GFAP is not detectable in all astrocytes but a common feature of reactive/activated astrocytes (Zamanian et al., 2012). We suggest astrocytic phagocytosis may be detrimentally increased to compensate for microglial dysfunction we proposed above (Konishi et al., 2020). However, we did not measure proteins, e.g., ATP-binding cassette transporter A1, which are better surrogate measures of astroglial phagocytosis (Morizawa et al., 2017), compared to GFAP-immunoreactivity used in this study.

While iron only treatment unexpectedly improved hippocampal function compared with iron-primed inflammation group (see above), iron appeared to have detrimental effects in the cortex as evidenced by increased cortical lipid peroxidation, compared with iron-primed inflammation. We suggest regional differences in the brain arise from variable iron requirements, with the cortex being relatively iron-sufficient compared to the hippocampus, the latter being particularly vulnerable to iron deficiency (Rao et al., 2013). Moreover, higher cortical oxygen consumption renders the cortex particularly vulnerable to lipid peroxidation (Nair et al., 1987). We propose greater cortical oxygen consumption alongside iron over-sufficiency in the cortex explains greater lipid peroxidation in the cortex compared with the hippocampus following iron treatment.

Increased FTL expression was observed in the brain cortex of mice exposed to LPS, with or without iron-priming. Ferritin consists of two subunits, FTH and FTL, which have distinct functions. Predominantly found in oligodendrocytes and neurons, FTH is a ferroxidase that oxidizes ferrous iron to ferric iron for storage. Microglia and astrocytes are equipped with FTL, which better sequestrates iron (Meadowcroft et al., 2015; Ashraf et al., 2018). Augmented microglial hyper-ramification and GFAP-immunoreactivity were observed only in the cortex of iron-primed mice subjected to LPS-induced inflammation, comparable to that in the hippocampus. Iron priming prior to subsequent mild inflammation in the cortex appears to induce a (functional) cellular iron overload prompting glial cells to increase FTL expression. Further evidence for apparently increased cellular iron is indicated by elevated IRP2 levels with iron-primed inflammation, but not with inflammation only. IRP2 expression has been shown to be increased in a 6-hydroxydopamine (6-OHDA)-model of Parkinson’s disease (PD), concomitant with increased iron accumulation (Jiang et al., 2010). In conditions of increased iron, a lack of IRP2 binding (signified by increased IRP2 levels) to the iron-responsive element (IRE) of ferritin mRNA leads to increased translation and expression of FTL (Rouault, 2006; Leipuviene and Theil, 2007). The apparent elevated cellular iron in iron + LPS mice compared to those treated with LPS only may arise from increased cellular iron import since we demonstrated elevated DMT1 in the former mice. DMT1 mediates both cellular iron uptake and endosomal iron export to increase cellular iron (Moos et al., 2007).

Notably, we show inflammation, with and without iron-priming, increased expression levels of cortical xCT, which has been demonstrated when System X_c_^−^ is inhibited (Yu et al., 2017). System X_c_^−^ comprises the widely expressed 4F2 heavy chain subunit, common to several amino acid transporters, and a specific xCT light chain subunit (Sato et al., 1999). Predominantly in glia, System X_c_^−^ is responsible for importing cystine in exchange for glutamate at the plasma membrane and is also called a cystine/glutamate antiporter (Sato et al., 1999; Gasol et al., 2004). Cystine is reduced to cysteine, the rate-limiting substrate for glutathione synthesis and crucial for GPX4 function. Inflammation-mediated inhibition of System X_c_^−^ may compromise cellular uptake of cystine, attenuate GPX4 activity, and increase susceptibility to oxidative stress. However, deletion of the xCT gene attenuated production of pro-inflammatory cytokines including nitric oxide, TNFα, and IL-6, while the expression of anti-inflammatory cytokines, e.g., Tm1/Chil3 were augmented, suggesting xCT also regulates microglial functions (Mesci et al., 2015).

Responses to iron and/or LPS-induced inflammation, not only differed in the hippocampus and cortex, but also in the basal ganglia regions, the striatum and substantia nigra. Mice exposed to LPS-induced inflammation, with or without iron-priming, showed decreased striatal ferroportin levels. Decreased ferroportin may be caused by increased hepcidin, a peptide hormone that enhances cellular degradation of ferroportin. Previously, systemic injection of hepcidin has been shown to decrease striatal ferroportin (Wang et al., 2010). Further, elevated striatal hepcidin has been observed under inflammatory conditions including ischemia and systemic bacterial inflammation (Ding et al., 2011; Lieblein-Boff et al., 2013). Increased degradation of ferroportin, the only known cellular iron exporter, attenuates iron egress and may lead to (functional) cellular iron overload in the striatum. LPS used in the present study originates from bacteria and can explain the concordance between our results and previous studies (Ding et al., 2011; Lieblein-Boff et al., 2013).

Elevated ACSL4 was also observed in iron + LPS mice in the striatum. ACSL4 catalyzes the insertion of arachidonic acid into phospholipids, specifically phosphatidylethanolamines. Phosphatidylethanolamines appear to be particularly susceptible to lipid peroxidation and the major lipid source for lipid peroxidation in ferroptosis and shown to be significant contributors to elevated 4-HNE adducts/lipid peroxidation products (Doll et al., 2017). Interestingly, mice with ACSL4-ablation, with limited ability to insert arachidonic acid into phosphatidylethanolamines, exhibited attenuated lipid peroxidation (Killion et al., 2018). The increased ACSL4 in response to iron-primed inflammation suggests increased susceptibility of the striatum to ferroptosis.

The alterations in ferroportin and ACSL4 in the striatum were not observed in the hippocampus and cortex. The striatum is known to have higher iron and microglial content than the cortex and hippocampus (Ramos et al., 2014; Ward et al., 2014; Grabert et al., 2016; Keller et al., 2018; Saba et al., 2020), which may explain the differential regional response. Further, GFAP-immunoreactivity was unaltered in the striatum but was increased in both the hippocampus and the cortex. This is unsurprising as astrocytes from the mouse striatum and hippocampus display diversity as confirmed by transcriptomic, proteomic, morphological, and functional evidence (Chai et al., 2017). Further, cortical astrocytes have been shown to release greater amounts of TNFα in response to LPS stimulation than striatal astrocytes (Saba et al., 2020), consistent with our findings of increased astroglial immunoreactivity in the cortex but not in the striatum.

Regional molecular comparisons were not possible with the substantia nigra as only metal and histological analyses were performed. However, we observed hyper-ramified microglia in the iron + LPS group compared with saline, iron, and LPS treatment groups. We suggest that iron activates microglia in the substantia nigra but revert to their usual surveillance mode unless exposed to a subsequent inflammatory episode. The substantia nigral neurons contain neuromelanin, which is known to sequester excess iron and inhibit free radical production (Zecca et al., 2008a). However, iron-loaded neuromelanin itself can be a source of redox-active iron to induce microgliosis and impair dopamine neuronal functioning (Faucheux et al., 2003; Zecca et al., 2008b; Zhang et al., 2011; Ward et al., 2014). A caveat of the study was that a comprehensive protein analysis of the substantia nigra to investigate iron dyshomeostasis and ferroptosis-like changes was not performed to allow comparison with other brain regions. Moreover, a functional readout of this anatomical region would have been useful to associate with molecular changes.

The total iron levels in the current study were unchanged despite iron treatment. A limitation of our study is that both spatial and bulk iron analysis by SRXRF and TXRF, respectively, measure all forms of iron and were used as proxy measures for the labile iron pool. The labile iron pool is relatively small compared to the total iron content and such iron measurements may not be sensitive/accurate to subtle fluctuations in the labile iron pool (Ashraf et al., 2019a, 2020). Ideally, future studies should directly measure changes in the labile iron pool.

We did not evaluate the mechanism of microglial and astrocytic activation in inflammation, but LPS has been shown to induce inflammation via crosstalk between microglia and astrocytes (Norris et al., 2005; Sama et al., 2008). Moreover, it is challenging to translate glial morphological plasticity to functionality. A limitation of the study is the lack of measurement of relevant pro- and anti-inflammatory cytokines in the brain and plasma that can be correlated with microglial priming. Additional functional readouts from *in vitro* experiments evaluating changes in microglial phenotype by flow cytometry may offer deeper mechanistic insights. To ascertain the propositions in this exploratory study, future studies are warranted to measure pro- and anti-inflammatory cytokines to determine their association with different microglial morphological states.

## Conclusion

We demonstrate inflammation following priming with systemic injections of mild iron doses in normal C57Bl/6J mice alters brain molecular profile suggestive of iron dyshomeostasis, lipid peroxidation, and neuroinflammation, in a region-dependent manner.

## Data availability statement

The original contributions presented in the study are included in the article/Supplementary material, further inquiries can be directed to the corresponding author.

## Ethics statement

The animal study was approved by local ethical review panel of King’s College London in accordance with the UK Home Office Animals Scientific Procedures Act 1986. The study was conducted in accordance with the local legislation and institutional requirements.

## Author contributions

AA: Conceptualization, Data curation, Formal analysis, Investigation, Methodology, Project administration, Visualization, Writing – original draft, Writing – review & editing. MA: Investigation, Writing – review & editing. CH: Investigation, Project administration, Writing – review & editing. JJ: Formal analysis, Investigation, Writing – review & editing. HP: Formal analysis, Investigation, Methodology, Writing – review & editing. KG: Formal analysis, Investigation, Methodology, Software, Writing – review & editing. AM: Formal analysis, Investigation, Writing – review & editing. AH: Funding acquisition, Resources, Writing – review & editing. P-WS: Conceptualization, Formal analysis, Funding acquisition, Investigation, Methodology, Project administration, Resources, Software, Supervision, Validation, Writing – original draft, Writing – review & editing.
