# Supplementary material for: Inflammation subsequent to mild iron excess differentially alters regional brain iron metabolism, oxidation and neuroinflammation status in mice
Source: Front Aging Neurosci. 2024 May 21;16:1393351. doi: 10.3389/fnagi.2024.1393351 (PMC11148467; doi:10.3389/fnagi.2024.1393351)
Supplement: Supplementary file 1 [file Data_Sheet_1.docx]

**Supplementary Material**

**Inflammation subsequent to mild iron excess differentially alters regional brain iron metabolism, oxidation and neuroinflammation status in mice**

**Supplementary Figures**

Figure S1: Nissl-stained coronal brain hemispheres from a C57Bl/6J mouse (Allen Mouse Brain Atlas, <https://mouse.brain-map.org>), showing region of interests manually drawn to delineate hippocampus (blue), cortex (green), striatum (pink) and substantia nigra (yellow) for synchrotron radiation X-ray fluorescence metal analysis.


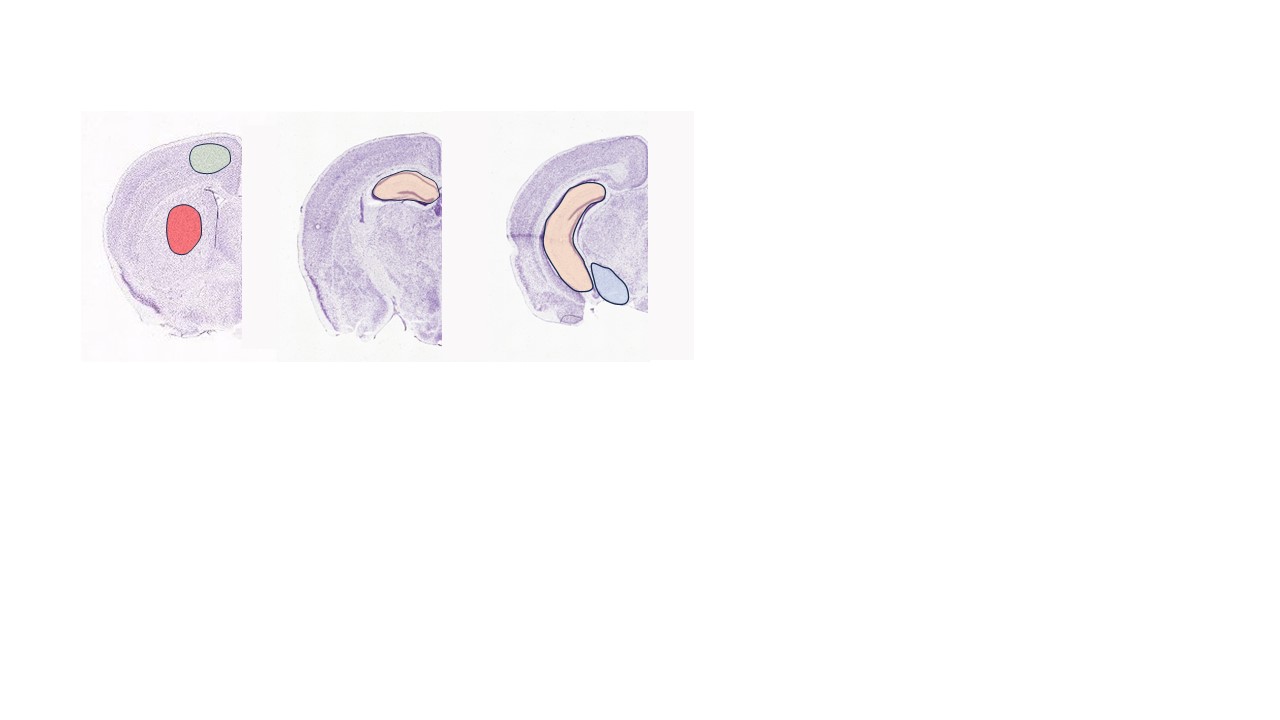


Image 52/132 Image 71/132 Image 83/132

**Supplementary Tables**

Table S1: Details of antibodies and dilutions, and blocking media (2% milk or BSA, PBS-Tween0.2%) used for western blotting. Appropriate secondary antibodies used included horseradish peroxidase (HRP)-conjugated anti-mouse, anti-rabbit, anti-rat, anti-goat, and anti-chicken secondary antibodies (62-6520, 31460, 712-035-150, PA1-28664, and SA1-72003, respectively; Thermofisher).

| **Antibody** | **Dilution** | **Blocking medium** |
| --- | --- | --- |
| Rabbit Ferritin light-chain (ab69090; Abcam) | 1/500 | Milk |
| Mouse Ferritin heavy-chain (MABC602; Merck) | 1/1000 | Milk |
| Mouse Transferrin receptor (13-6800; Thermofisher) | 1/1000 | BSA |
| Rabbit Divalent metal transporter 1 (ab123085; Abcam) | 1/1000 | Milk |
| Rabbit Iron Responsive Element Binding Protein 2 (PA1-16543; Thermofisher) | 1/500 | BSA |
| Rabbit Ceruloplasmin (PA5-14225; Thermofisher) | 1/500 | Milk |
| Rabbit Ferroportin (ab78066; Abcam) | 1/1000 | BSA |
| Rabbit Heme-oxygenase 1 (ab1284; Merck) | 1/1000 | Milk |
| Goat Iba1 (SAB2500042, Merck) | 1/500 | Milk |
| Chicken GFAP (ab134436, Abcam) | 1/15000 | Milk |
| Rabbit NADPH oxidase 2 (ab80508; Abcam) | 1/1000 | Milk |
| Rat Triggering receptor expressed on myeloid cells 2  (ab86491; Abcam) | 1/500 | Milk |
| Rabbit Acyl-CoA Synthetase Long-Chain Family Member 4 (ab155282; Abcam) | 1/10000 | Milk |
| Goat light-chain subunit of system X_c_^-^ (xCT; SAB2500951; Merck) | 1/500 | Milk |
| Rabbit Glutathione peroxidase 4 (SAB4300725; Merck) | 1/500 | Milk |
| Rabbit 4-Hydroxynonenal (ab46545; Abcam) | 1/1000 | Milk |
| Mouse HRP-conjugated β-actin (clone AC-15, ab49900; Abcam) | 1/25000 | Milk |

Table S2: Acute (two-days post-dose) effects of mild systemic iron treatment on iron regulatory, ferroptosis and glia proteins; metals; and glia morphology in the hippocampus (Cohort 1: saline- or iron-treated [n=5/group] mice injected daily for five days were killed two days after the final injection and brains isolated to study the acute effects of mild iron treatment on the brain). (Note that acyl-CoA synthetase long chain fatty acid family member 4 was not detectable.) Two-tailed Student’s unpaired t-test was used to examine differences between saline and iron groups, with p < 0.05 being significant (in bold) but did not remain significant after FDR-correction. (Abbreviations: ferritin light-chain, FTL; ferritin heavy-chain, FTH; heme oxygenase-1, HO-1; iron regulatory protein 2, IRP2; transferrin receptor, TfR; divalent metal transporter 1, DMT1; ceruloplasmin, Cp; ferroportin, Fpn; light-chain subunit of System X_c_^-^, xCT; 4-hydroxynonenal, 4-HNE; glutathione peroxidase 4, GPX4; NADPH oxidase 2, NOX2; ionized calcium-binding adaptor molecule 1, Iba1; glial fibrillary acidic protein, GFAP; triggering receptor expressed on myeloid cells 2, TREM2; endpoints, EP; branch length, BL; signal intensity, SI; arbitrary units, a.u.).

| **Hippocampus** | | | | | |
| --- | --- | --- | --- | --- | --- |
| **Proteins / Metals** | **Method** | **Saline Group** | **Iron Group** | **t(df)** | **p-value** |
| FTL | Western blot | 0.29±0.06 | 0.219±0.044 | 2.195(7.600) | 0.0612 |
| FTH |  | 1.09±0.71 | 1.25±0.75 | 0.349(7.970) | 0.7360 |
| HO1 |  | 0.24±0.12 | 0.20±0.03 | 0.601(4.585) | 0.5765 |
| IRP2 |  | 5.53±1.75 | 5.18±1.18 | 0.374(7.002) | 0.7196 |
| TfR |  | 5.44±3.29 | 5.97±3.35 | 0.253(7.997) | 0.8067 |
| DMT1 |  | 1.08±0.45 | 0.70±0.22 | 1.713(5.903) | 0.1384 |
| Cp |  | 1.19±0.32 | 1.02±0.13 | 1.110(5.353) | 0.3145 |
| Fpn |  | 0.13±0.05 | 0.13±0.05 | 0.111(7.912) | 0.9146 |
| xCT |  | 0.97±0.52 | 0.92±0.24 | 0.175(5.583) | 0.8675 |
| 4-HNE |  | 0.47±0.04 | 0.39±0.05 | 2.613(7.918) | **0.0313** |
| GPX4 |  | 1.62±0.41 | 1.90±1.08 | 0.540(5.137) | 0.6121 |
| NOX2 |  | 1.21±1.07 | 2.61±2.87 | 1.023(5.089) | 0.3524 |
| Iba1 |  | 3.63±0.65 | 3.54±0.59 | 0.223(7.913) | 0.8295 |
| GFAP |  | 4.62±1.69 | 3.94±0.91 | 0.799(6.161) | 0.4541 |
| TREM2 |  | 0.14±0.06 | 0.17±0.06 | 0.908(4.363) | 0.4113 |
| Microglial EP/cell | Immunohisto-chemistry | 29.30±6.09 | 34.40±4.03 | 1.562(6.933) | 0.1626 |
| Microglial BL/cell (µm) |  | 581.60±164.30 | 539.88±137.63 | 0.435(7.762) | 0.6753 |
| GFAP-SI (a.u.) |  | 27.34±2.90 | 24.69±2.78 | 1.478(7.986) | 0.1777 |
| Iron (mg/kg) | Total reflection  X-ray fluorescence | 279.79±56.69 | 293.06±53.64 | 0.380(7.976) | 0.7135 |
| Zinc (mg/kg) |  | 387.08±72.00 | 377.49±60.93 | 0.228(7.787) | 0.8258 |
| Copper (mg/kg) |  | 118.04±18.04 | 109.78±15.46 | 0.779(7.821) | 0.4590 |

Table S3: Acute (two-days post-dose) effects of mild systemic iron treatment on iron regulatory, ferroptosis and glia proteins; metals; and glia morphology in the cortex (Cohort 1: saline- or iron-treated [n=5/group] mice injected daily for five days were killed two days after the final injection and brains isolated to study the acute effects of mild iron treatment on the brain). (Note that acyl-CoA synthetase long chain fatty acid family member 4 was not detectable.) Two-tailed Student’s unpaired t-test was used to examine differences between saline and iron groups, with p < 0.05 being significant (in bold), but they did not remain significant after FDR-correction. (Abbreviations: ferritin light-chain, FTL; ferritin heavy-chain, FTH; heme oxygenase-1, HO-1; iron regulatory protein 2, IRP2; transferrin receptor, TfR; divalent metal transporter 1, DMT1; ceruloplasmin, Cp; ferroportin, Fpn; light-chain subunit of System X_c_^-^, xCT; 4-hydroxynonenal, 4-HNE; glutathione peroxidase 4, GPX4; NADPH oxidase 2, NOX2; ionized calcium-binding adaptor molecule 1, Iba1; glial fibrillary acidic protein, GFAP; triggering receptor expressed on myeloid cells 2, TREM2; endpoints, EP; branch length, BL; signal intensity, SI; arbitrary units, a.u.).

| **Cortex** | | | | | |
| --- | --- | --- | --- | --- | --- |
| **Proteins / Metals** | **Method** | **Saline Group** | **Iron Group** | **t(df)** | **p-value** |
| FTL | Western blot | 0.91±0.23 | 0.83±0.43 | 0.376(6.088) | 0.7200 |
| FTH |  | 0.38±0.12 | 0.39±0.13 | 0.139(7.957) | 0.8933 |
| HO-1 |  | 0.62±0.16 | 0.43±0.08 | 2.338(5.742) | 0.0599 |
| IRP2 |  | 4.19±1.81 | 2.50±0.84 | 1.890±5.630 | 0.1108 |
| TfR |  | 4.46±0.43 | 3.51±0.72 | 2.545(6.541) | **0.0406** |
| DMT1 |  | 1.94±0.54 | 1.65±0.54 | 0.847(8.000) | 0.4214 |
| Cp |  | 0.56±0.18 | 0.40±0.11 | 1.677(6.641) | 0.1397 |
| Fpn |  | 0.95±0.22 | 1.12±0.25 | 1.107(7.846) | 0.3012 |
| xCT |  | 0.43±0.12 | 0.37±0.11 | 0.881(7.881) | 0.4042 |
| 4-HNE |  | 1.16±0.20 | 1.13±0.14 | 0.303(7.222) | 0.7708 |
| GPX4 |  | 2.66±0.15 | 2.49±0.49 | 0.731(4.763) | 0.4993 |
| NOX2 |  | 3.88±1.88 | 4.06±1.57 | 0.170(7.757) | 0.8694 |
| Iba1 |  | 4.24±1.09 | 4.33±0.67 | 0.154(6.632) | 0.8820 |
| GFAP |  | 1.71±0.40 | 1.38±0.48 | 1.185(7.758) | 0.2710 |
| TREM2 |  | 2.83±0.86 | 1.74±0.32 | 2.670(5.123) | 0.0433 |
| Microglial EP/cell | Immunohisto-chemistry | 28.71±8.24 | 31.73±6.29 | 0.650(7.479) | 0.5352 |
| Microglial BL/cell (µm) |  | 556.45±189.02 | 565.17±205.21 | 0.070(7.947) | 0.9460 |
| GFAP-SI (a.u.) |  | 29.03±4.31 | 25.94±1.97 | 1.456(5.601) | 0.1991 |
| Iron (mg/kg) | Total reflection  X-ray fluorescence | 337.14±43.46 | 321.42±70.34 | 0.425(6.665) | 0.6841 |
| Zinc (mg/kg) |  | 342.50±53.17 | 372.68±118.13 | 0.521(5.557) | 0.6225 |
| Copper (mg/kg) |  | 138.76±9.45 | 102.24±27.60 | 2.799(4.925) | 0.0387 |

Table S4: Acute (two-days post-dose) effects of mild systemic iron treatment on iron regulatory, ferroptosis and glia proteins; metals; and glia morphology in the striatum (Cohort 1: saline- or iron-treated [n=5/group] mice injected daily for five days were killed two days after the final injection and brains isolated to study the acute effects of mild iron treatment on the brain). Two-tailed Student’s unpaired t-test was used to examine differences between saline and iron groups, with p < 0.05 being significant. (Abbreviations: ferritin light-chain, FTL; ferritin heavy-chain, FTH; heme oxygenase-1, HO-1; iron regulatory protein 2, IRP2; transferrin receptor, TfR; divalent metal transporter 1, DMT1; ceruloplasmin, Cp; ferroportin, Fpn; light-chain subunit of System X_c_^-^, xCT; acyl-CoA synthetase long chain fatty acid family member 4, ACSL4; 4-hydroxynonenal, 4-HNE; glutathione peroxidase 4, GPX4; NADPH oxidase 2, NOX2; ionized calcium-binding adaptor molecule 1, Iba1; glial fibrillary acidic protein, GFAP; triggering receptor expressed on myeloid cells 2, TREM2; endpoints, EP; branch length, BL; signal intensity, SI; arbitrary units, a.u.).

| **Striatum** | | | | | |
| --- | --- | --- | --- | --- | --- |
| **Protein / Metals** | **Method** | **Saline Group** | **Iron Group** | **t(df)** | **p-value** |
| FTL | Western blot | 0.55±0.24 | 0.52±0.15 | 0.220(6.633) | 0.8323 |
| FTH |  | 1.10±0.19 | 1.11±0.18 | 0.028(7.995) | 0.9783 |
| HO-1 |  | 0.47±0.07 | 0.39±0.18 | 0.956(5.329) | 0.3805 |
| IRP2 |  | 1.09±0.22 | 0.80±0.23 | 1.444(7.989) | 0.1868 |
| TfR |  | 2.98±2.35 | 1.81±1.10 | 1.009(5.663) | 0.3542 |
| DMT1 |  | 1.78±0.35 | 1.40±0.18 | 2.137(5.955) | 0.0768 |
| Cp |  | 2.13±0.54 | 1.65±0.67 | 1.267(7.664) | 0.2422 |
| Fpn |  | 1.02±0.35 | 0.84±0.25 | 0.942(7.107) | 0.3770 |
| xCT |  | 0.96±0.17 | 0.78±0.14 | 1.806(7.733) | 0.1099 |
| ACSL4 |  | 2.68±1.50 | 3.45±0.28 | 1.131(4.274) | 0.3173 |
| 4-HNE |  | 1.38±0.55 | 1.33±0.64 | 0.136(7.853) | 0.8951 |
| GPX4 |  | 1.81±0.27 | 1.74±0.33 | 0.388(7.699) | 0.7083 |
| NOX2 |  | 0.78±0.29 | 0.50±0.10 | 2.102(5.042) | 0.0891 |
| Iba1 |  | 17.37±8.74 | 17.65±4.73 | 0.063(6.157) | 0.9517 |
| GFAP |  | 0.80±0.14 | 1.00±0.31 | 1.320(5.549) | 0.2388 |
| TREM2 |  | 0.36±0.11 | 0.31±0.06 | 0.830(6.113) | 0.4379 |
| Microglial EP/cell | Immunohisto-chemistry | 31.77±8.50 | 36.13±10.94 | 0.704(7.539) | 0.5027 |
| Microglial BL/cell (µm) |  | 592.06±141.41 | 581.51±185.54 | 0.101(7.475) | 0.9221 |
| GFAP-SI (a.u.) |  | 23.81±5.45 | 16.73±4.66 | 2.208(7.812) | 0.0590 |
| Iron (mg/kg) | Total reflection  X-ray Fluorescence | 210.64±71.40 | 258.44±106.03 | 0.836(7.009) | 0.4307 |
| Zinc (mg/kg) |  | 197.80±52.32 | 234.44±73.61 | 0.907(7.219) | 0.3936 |
| Copper (mg/kg) |  | 102.52±19.36 | 92.14±31.62 | 0.626(6.629) | 0.5522 |

Table S5: The effects of acute iron treatments (two-days post-injection) in the substantia nigra (Cohort 1: saline- or iron-treated [n=5/group] mice injected daily for five days were killed two days after the final injection and brains isolated to study the acute effects of mild iron treatment on the brain) on microglial morphology and GFAP-immunoreactivity. Differences between saline and iron groups were assessed by two-tailed Student’s unpaired t-test with p < 0.05 being significant. (Abbreviations: glial fibrillary acidic protein, GFAP).

| **Assessment** | **Saline Group** | **Iron Group** | **t(df)** | **p-value** |
| --- | --- | --- | --- | --- |
| Microglial endpoints/cell | 41.0±4.8 | 36.2±5.1 | 1.454(6.743) | 0.1908 |
| Microglial branch length/cell (µm) | 457.41±144.22 | 513.74±210.85 | 0.475(6.900) | 0.6498 |
| GFAP-signal intensity (arbitrary units) | 28.95±4.73 | 27.63±3.98 | 0.476(7.777) | 0.6474 |

Table S6: Chronic (three-weeks post-dose) effects of mild systemic iron treatment on iron regulatory, ferroptosis and glia proteins; metals; and glia morphology in the hippocampus (Cohort 2: saline [n=7] and iron-treated [n=8] mice were killed at three weeks post-dose [aged 12 weeks] and brains isolated to determine the potential longer term/chronic effect of a single iron treatment). (Note that acyl-CoA synthetase long chain fatty acid family member 4 was not detectable.) Two-tailed student’s unpaired t-test was used to examine differences between saline and iron groups, with p < 0.05 being significant. Note, for synchrotron radiation X-ray fluorescence (SRXRF) mapping, only n = 4/group were analyzed. Abbreviations: ferritin light-chain, FTL; ferritin heavy-chain, FTH; heme oxygenase-1, HO-1; iron regulatory protein 2, IRP2; transferrin receptor, TfR; divalent metal transporter 1, DMT1; ceruloplasmin, Cp; ferroportin, Fpn; light-chain subunit of System X_c_^-^, xCT; 4-hydroxynonenal, 4-HNE; glutathione peroxidase 4, GPX4; NADPH oxidase 2, NOX2; ionized calcium-binding adaptor molecule 1, Iba1; glial fibrillary acidic protein, GFAP; triggering receptor expressed on myeloid cells 2, TREM2; endpoints, EP; branch length, BL; signal intensity, SI; arbitrary units, a.u.).

| **Hippocampus** | | | | | |
| --- | --- | --- | --- | --- | --- |
| **Protein / Metals** | **Method** | **Saline Group** | **Iron Group** | **t(df)** | **p-value** |
| FTL | Western blot | 0.14±0.04 | 0.13±0.04 | 0.510(11.90) | 0.6192 |
| FTH |  | 2.28±1.12 | 1.56±0.69 | 1.453(9.988) | 0.1769 |
| HO-1 |  | 0.39±0.28 | 0.28±0.18 | 0.787(9.976) | 0.4497 |
| IRP2 |  | 5.90±0.96 | 5.04±0.93 | 1.706(11.99) | 0.1138 |
| TfR |  | 1.54±0.44 | 1.28±0.33 | 1.271(11.23) | 0.2293 |
| DMT1 |  | 1.64±0.66 | 1.30±0.30 | 1.238(8.449) | 0.2491 |
| Cp |  | 0.57±0.23 | 0.60±0.20 | 0.255(11.79) | 0.8029 |
| Fpn |  | 0.10±0.03 | 0.13±0.08 | 1.015(7.772) | 0.3406 |
| xCT |  | 0.63±0.17 | 0.71±0.15 | 0.918(10.22) | 0.3796 |
| 4-HNE |  | 0.98±0.59 | 0.95±0.48 | 0.128(11.55) | 0.9008 |
| GPX4 |  | 4.75±0.21 | 4.28±0.83 | 1.462(6.781) | 0.1884 |
| NOX2 |  | 0.26±0.11 | 0.37±0.05 | 2.211(6.834) | 0.0636 |
| Iba1 |  | 3.58±1.16 | 3.67±0.61 | 0.191(9.057) | 0.8529 |
| GFAP |  | 2.71±0.80 | 2.22±0.65 | 1.250(11.56) | 0.2360 |
| TREM2 |  | 0.17±0.06 | 0.20±0.07 | 0.917(7.806) | 0.3868 |
| Microglial EP/cell | Immunohisto-chemistry | 35.16±2.42 | 33.46±2.01 | 1.394(9.664) | 0.1945 |
| Microglial BL/cell (µm) |  | 392..27±17.71 | 410.60±38.13 | 1.198(10.40) | 0.2576 |
| GFAP-SI (a. u.) |  | 25.64±4.42 | 28.92±3.02 | 1.567(8.377) | 0.1541 |
| Iron (mg/kg) | Total reflection X-ray fluorescence | 28.67±7.14 | 26.79±5.91 | 0.552(11.73) | 0.5912 |
| Zinc (mg/kg) |  | 39.71±5.73 | 37.35±13.48 | 0.452(9.703) | 0.6615 |
| Copper (mg/kg) |  | 14.66±1.37 | 13.16±2.41 | 1.501(11.31) | 0.1607 |
| Iron (mg/kg) | SRXRF | 24.83±1.02 | 25.06±0.81 | 0.356(5.710) | 0.7347 |
| Zinc (mg/kg) |  | 26.99±1.18 | 27.14±0.64 | 0.222(4.630) | 0.8342 |
| Copper (mg/kg) |  | 9.80±0.16 | 9.94±0.17 | 1.170(5.989) | 0.2864 |

Table S7: Chronic (three weeks post-dose) effects of mild systemic iron treatment on iron regulatory, ferroptosis and glia proteins; metals; and glia morphology in the cortex (Cohort 2: saline [n=7] and iron-treated [n=8] mice were killed at three weeks post-dose [aged 12 weeks] and brains isolated to determine the potential longer term/chronic effect of a single iron treatment). (Note that acyl-CoA synthetase long chain fatty acid family member 4 was not detectable.) Two-tailed Student’s unpaired t-test was used to examine differences between saline and iron groups, with p < 0.05 being significant. Note, for synchrotron radiation X-ray fluorescence mapping (SRXRF), only n = 4/group were analyzed. (Abbreviations: ferritin light-chain, FTL; ferritin heavy-chain, FTH; heme oxygenase-1, HO-1; iron regulatory protein 2, IRP2; transferrin receptor, TfR; divalent metal transporter 1, DMT1; ceruloplasmin, Cp; ferroportin, Fpn; light-chain subunit of System X_c_^-^, xCT; 4-hydroxynonenal, 4-HNE; glutathione peroxidase 4, GPX4; NADPH oxidase 2, NOX2; ionized calcium-binding adaptor molecule 1, Iba1; glial fibrillary acidic protein, GFAP; triggering receptor expressed on myeloid cells 2, TREM2; endpoints, EP; branch length, BL; signal intensity, SI; arbitrary units, a.u.).

| **Cortex** | | | | | |
| --- | --- | --- | --- | --- | --- |
| **Protein / Metals** | **Method** | **Saline** | **Iron** | **t(df)** | **p-value** |
| FTL | Western blot | 0.36±0.14 | 0.50±0.27 | 1.212(10.86) | 0.2511 |
| FTH |  | 0.87±0.36 | 1.03±0.34 | 0.825(11.97) | 0.4253 |
| HO-1 |  | 0.46±0.13 | 0.50±0.25 | 0.350(10.67) | 0.7330 |
| IRP2 |  | 2.40±1.42 | 2.37±1.50 | 0.034(9.061) | 0.9736 |
| TfR |  | 1.04±0.27 | 1.39±0.61 | 1.446(10.19) | 0.1782 |
| DMT1 |  | 2.48±0.54 | 2.73±1.23 | 0.530(9.833) | 0.6078 |
| Cp |  | 0.63±0.15 | 0.70±0.13 | 1.035(12.01) | 0.3210 |
| Fpn |  | 0.43±0.09 | 0.44±0.12 | 0.124(12.75) | 0.9032 |
| xCT |  | 3.65±1.59 | 3.49±1.54 | 0.195(11.99) | 0.8485 |
| 4-HNE |  | 1.06±0.14 | 1.22±0.20 | 1.915(12.59) | 0.0785 |
| GPX4 |  | 2.65±0.65 | 2.76±1.05 | 0.246(11.80) | 0.8102 |
| NOX2 |  | 1.01±0.17 | 1.29±0.52 | 1.417(8.813) | 0.1909 |
| Iba1 |  | 6.15±1.43 | 5.96±1.20 | 0.277(11.79) | 0.7866 |
| GFAP |  | 2.33±2.19 | 1.56±0.78 | 0.904(7.408) | 0.3945 |
| TREM2 |  | 0.51±0.19 | 0.50±0.17 | 0.151(12.27) | 0.8823 |
| Microglial EP/cell | Immunohisto-chemistry | 34.06±3.48 | 33.69±2.29 | 0.239(10.15) | 0.8156 |
| Microglial BL/cell (µm) |  | 389.80±48.99 | 430.59±57.39 | 1.485(13.00) | 0.1615 |
| GFAP SI (a.u.) |  | 26.55±5.72 | 28.55±3.79 | 0.786(10.20) | 0.4497 |
| Iron (mg/kg) | Total reflection  X-ray  fluorescence | 110.43±174.83 | 40.83±11.30 | 1.051(6.044) | 0.3333 |
| Zinc (mg/kg) |  | 55.80±13.79 | 56.25±16.00 | 0.059(13.00) | 0.9542 |
| Copper (mg/kg) |  | 18.14±2.47 | 17.10±2.67 | 0.785(12.94) | 0.4464 |
| Iron (mg/kg) | SRXRF | 24.49±1.64 | 22.18±3.30 | 1.255(4.388) | 0.2721 |
| Zinc (mg/kg) |  | 26.12±3.61 | 21.53±4.70 | 1.552(5.626) | 0.1748 |
| Copper (mg/kg) |  | 9.80±0.53 | 9.25±0.87 | 1.085(4.960) | 0.3280 |

Table S8: Chronic (three weeks post-dose) effects of mild systemic iron treatment on iron regulatory, ferroptosis and glia proteins; metals; and glia morphology in the striatum (Cohort 2: saline [n=7] and iron-treated [n=8] mice were killed at three weeks post-dose [aged 12 weeks] and brains isolated to determine the potential longer term/chronic effect of a single iron treatment). Two-tailed Student’s unpaired t-test was used to examine differences between saline and iron groups, with p < 0.05 being significant. Note, for synchrotron radiation X-ray fluorescence mapping (SRXRF), only n = 4/group were analyzed. (Abbreviations: ferritin light-chain, FTL; ferritin heavy-chain, FTH; heme oxygenase-1, HO-1; iron regulatory protein 2, IRP2; transferrin receptor, TfR; divalent metal transporter 1, DMT1; ceruloplasmin, Cp; ferroportin, Fpn; light-chain subunit of System X_c_^-^, xCT; acyl-CoA synthetase long chain fatty acid family member 4, ACSL4; 4-hydroxynonenal, 4-HNE; glutathione peroxidase 4, GPX4; NADPH oxidase 2, NOX2; ionized calcium-binding adaptor molecule 1, Iba1; glial fibrillary acidic protein, GFAP; triggering receptor expressed on myeloid cells 2, TREM2; endpoints, EP; branch length, BL; signal intensity, SI; arbitrary units, a.u.).

| **Striatum** | | | | | |
| --- | --- | --- | --- | --- | --- |
| **Protein / Metals** | **Method** | **Saline Group** | **Iron Group** | **t(df)** | **p-value** |
| FTL | Western blot | 0.41±0.14 | 0.51±0.19 | 1.143(12.80) | 0.2740 |
| FTH |  | 0.48±0.19 | 0.80±0.57 | 1.529(8.770) | 0.1614 |
| HO-1 |  | 0.53±0.13 | 0.58±0.17 | 0.570(11.39) | 0.5800 |
| IRP2 |  | 0.94±0.30 | 1.07±0.42 | 0.716(12.00) | 0.4876 |
| TfR |  | 0.77±0.38 | 0.56±0.23 | 1.275(9.705) | 0.2319 |
| DMT1 |  | 1.57±0.18 | 1.62±0.38 | 0.345(10.31) | 0.7371 |
| Cp |  | 0.34±0.05 | 0.36±0.08 | 0.470(10.03) | 0.6485 |
| Fpn |  | 0.43±0.10 | 0.54±0.32 | 0.903(7.153) | 0.3960 |
| xCT |  | 0.50±0.04 | 0.66±0.26 | 1.775(7.452) | 0.1166 |
| ACSL4 |  | 2.45±0.61 | 2.23±0.77 | 0.615(11.44) | 0.5508 |
| 4-HNE |  | 0.30±0.11 | 0.53±0.35 | 1.815(8.594) | 0.1045 |
| GPX4 |  | 1.71±0.52 | 1.54±0.25 | 0.806(8.678) | 0.4418 |
| NOX2 |  | 0.54±0.09 | 0.65±0.09 | 2.156(10.82) | 0.0545 |
| Iba1 |  | 7.31±1.13 | 8.43±1.68 | 1.532(12.28) | 0.1508 |
| GFAP |  | 1.12±0.40 | 1.04±0.44 | 0.331(11.40) | 0.7465 |
| TREM2 |  | 0.48±0.13 | 0.55±0.17 | 1.006(12.80) | 0.3333 |
| Microglial EP/cell | Immunohisto  -chemistry | 39.56±3.79 | 36.44±2.51 | 1.751(8.204) | 0.1172 |
| Microglial BL/cell (µm) |  | 455.30±25.15 | 420.35±64.41 | 1.400(9.580) | 0.1932 |
| GFAP SI (a.u.) |  | 19.00±5.51 | 23.14±9.15 | 1.026(9.842) | 0.3294 |
| Iron (mg/kg) | Total reflection  X-ray fluorescence | 108.07±219.55 | 44.88±34.63 | 0.753(6.262) | 0.4786 |
| Zinc (mg/kg) |  | 25.83±13.33 | 23.28±16.85 | 0.327(12.90) | 0.7487 |
| Copper (mg/kg) |  | 13.76±2.17 | 14.05±5.35 | 0.142(9.494) | 0.8901 |
| Iron (mg/kg) | SRXRF | 29.95±1.84 | 29.03±4.03 | 0.417(4.194) | 0.6972 |
| Zinc (mg/kg) |  | 24.98±2.01 | 25.55±2.32 | 0.370(5.883) | 0.7243 |
| Copper (mg/kg) |  | 10.83±0.48 | 10.83±0.50 | 0.015(6.000) | 0.9881 |

Table S9: Chronic (three weeks post-dose) effects of mild systemic iron treatment on glia morphology and metals in the substantia nigra (Cohort 2: saline [n=7] and iron-treated [n=8] mice were killed at three weeks post-dose [aged 12 weeks] and brains isolated to determine the potential longer term/chronic effect of a single iron treatment). Two-tailed Student’s unpaired t-test was used to examine differences between saline and iron groups, with p < 0.05 being significant (in bold), but they did not remain significant after FDR-correction. Note, for synchrotron radiation X-ray fluorescence mapping (SRXRF), only n = 4/group were analyzed. (Abbreviations: arbitrary units, a.u.)

| **Substantia Nigra** | | | | | |
| --- | --- | --- | --- | --- | --- |
| **Glia / Metals** | **Method** | **Saline Group** | **Iron Group** | **t(df)** | **p-value** |
| Microglial endpoints/cell | Immuno-  histo-chemistry | 34.90±1.31 | 32.00±5.63 | 1.282(7.256) | 0.2394 |
| Microglial branch length/cell (µm) |  | 426.41±20.48 | 353.30±49.49 | 3.304(7.947) | **0.0109** |
| GFAP signal intensity (a.u.) |  | 26.79±7.37 | 30.11±3.71 | 0.842(3.889) | 0.4485 |
| Iron (mg/kg) | SRXRF | 32.93±3.26 | 32.61±1.99 | 0.164(4.965) | 0.8761 |
| Zinc (mg/kg) |  | 25.28±2.88 | 25.74±1.72 | 0.278(4.902) | 0.7927 |
| Copper (mg/kg) |  | 9.50±0.77 | 9.57±0.35 | 0.186(4.214) | 0.8613 |

Table S10: The effects of mild systemic iron and/or lipopolysaccharide (LPS)-treatments on iron regulatory, ferroptosis and glia proteins; metals; and glia morphology in the hippocampus (Cohort 3: saline only [control group, n=7], iron only [n=8], LPS only [n=8] and iron+LPS group [n=8]). (Note that acyl-CoA synthetase long chain fatty acid family member 4 was not detectable.) One-way ANOVA and *post hoc* Tukey correction to determine significant differences between saline, iron, LPS and iron+LPS groups. P ≤ 0.05 was considered significantly different from saline^a^, iron^b^, and LPS^c^ *post hoc* Tukey correction (in bold). (Abbreviations: ferritin light-chain, FTL; ferritin heavy-chain, FTH; heme oxygenase-1, HO-1; iron regulatory protein 2, IRP2; transferrin receptor, TfR; divalent metal transporter 1, DMT1; ceruloplasmin, Cp; ferroportin, Fpn; light-chain subunit of System X_c_^-^, xCT; 4-hydroxynonenal, 4-HNE; glutathione peroxidase 4, GPX4; NADPH oxidase 2, NOX2; ionized calcium-binding adaptor molecule 1, Iba1; glial fibrillary acidic protein, GFAP; triggering receptor expressed on myeloid cells 2, TREM2; endpoints, EP; branch length, BL; signal intensity, SI; arbitrary units, a.u.).

| **Hippocampus** | | | | | | |
| --- | --- | --- | --- | --- | --- | --- |
| **Protein / metal** | **Saline Group** | **Iron Group** | **LPS Group** | **Iron+LPS Group** | **F(DFn,DFd)** | **p-value** |
| **Western blotting** | | | | | | |
| FTL | 1.44±0.23 | 1.53±0.55 | 1.76±0.75 | 1.57±0.40 | 0.442(3,23) | 0.7255 |
| FTH | 0.18±0.03 | 0.17±0.08 | 0.17±0.08 | 0.17±0.02 | 0.014(3,21) | 0.9977 |
| HO-1 | 0.17±0.03 | 0.13±0.02 | 0.11±0.02^a^ | 0.11±0.04^a^ | 5.732(3,20) | **0.0053** |
| IRP2 | 1.43±0.67 | 1.76±0.91 | 1.32±0.34 | 1.10±0.33 | 1.148(3,22) | 0.3520 |
| TfR | 0.78±0.23 | 0.59±0.17 | 0.64±0.16 | 0.57±0.13 | 1.854(3,23) | 0.1656 |
| DMT1 | 0.49±0.14 | 0.52±0.25 | 0.50±0.20 | 0.64±0.40 | 0.500(3,27) | 0.6853 |
| Cp | 0.93±0.08 | 1.04±0.27 | 1.09±0.27 | 1.05±0.27 | 0.475(3,26) | 0.7021 |
| Fpn | 0.16±0.08 | 0.18±0.07 | 0.17±0.09 | 0.15±0.08 | 0.132(3,27) | 0.9403 |
| xCT | 0.87±0.20 | 0.87±0.16 | 0.84±0.19 | 0.89±0.26 | 0.070(3,27) | 0.9755 |
| 4-HNE | 0.62±0.19 | 0.59±0.13 | 0.91±0.23^a,b^ | 0.92±0.19^a,b^ | 6.400(3,23) | **0.0026** |
| GPX4 | 0.23±0.11 | 0.32±0.12 | 0.35±0.12 | 0.35±0.14 | 1.679(3,24) | 0.1980 |
| NOX2 | 0.22±0.07 | 0.27±0.12 | 0.28±0.09 | 0.34±0.16 | 1.142(3,22) | 0.3540 |
| Iba1 | 0.14±0.02 | 0.16±0.02 | 0.22±0.06^a,b^ | 0.17±0.02 | 6.057(3,19) | **0.0045** |
| GFAP | 0.85±0.18 | 0.74±0.09 | 1.24±0.25^a,b^ | 1.02±0.23 | 6.171(3,20) | **0.0038** |
| TREM2 | 0.43±0.10 | 0.59±0.12 | 0.64±0.14^a^ | 0.35±0.17^b,c^ | 6.631(3,22) | **0.0023** |
| **Immunohistochemistry** | | | | | | |
| Microglial EP/cell | 40.60±7.30 | 37.47±3.77 | 35.76±2.58 | 37.28±3.68 | 1.454(3,27) | 0.2493 |
| Microglial BL/cell | 276.46±29.51 | 269.36±29.56 | 303.63±41.47 | 387.40±116.41^a,b^ | 5.278(3,27) | **0.0054** |
| GFAP SI (a.u.) | 34.36±2.05 | 35.74±2.77 | 37.60±2.90 | 39.48±1.94^a,b^ | 6.221(3,27) | **0.0024** |
| **Total reflection X-ray fluorescence** | | | | | | |
| Iron (mg/kg) | 30.51±6.06 | 29.99±8.77 | 25.03±5.72 | 27.21±4.54 | 1.094(3,24) | 0.3706 |
| Zinc (mgk/g) | 36.49±6.20 | 38.24±5.20 | 33.07±5.02 | 38.70±5.75 | 1.477(3,24) | 0.2459 |
| Copper (mg/kg) | 14.17±1.65 | 14.61±2.32 | 13.17±1.91 | 14.01±1.58 | 0.716(3,24) | 0.5521 |
| **Synchrotron radiation X-ray fluorescence** | | | | | | |
| Iron (mg/kg) | 46.64±8.60 | 46.52±5.28 | 44.09±7.22 | 46.02±8.87 | 0.191(3,27) | 0.9019 |
| Zinc (mg/kg) | 55.72±11.55 | 55.71±6.69 | 51.86±7.44 | 54.84±12.70 | 0.269(3,27) | 0.8469 |
| Copper (mg/kg) | 49.45±9.29 | 49.85±5.02 | 47.39±6.36 | 47.95±8.89 | 0.193(3,27) | 0.9003 |

Table S11: The effects of mild systemic iron and/or lipopolysaccharide (LPS)-treatments on iron regulatory, ferroptosis and glia proteins; metals; and glia morphology in the cortex (Cohort 3: saline only [control group, n=7], iron only [n=8], LPS only [n=8] and iron+LPS group [n=8]). (Note that acyl-CoA synthetase long chain fatty acid family member 4 was not detectable.) One-way ANOVA and *post hoc* Tukey correction to determine significant differences between saline, iron, LPS and iron+LPS groups. P ≤ 0.05 was considered significant different from saline^a^, iron^b^ and LPS^c^ *post hoc* Tukey correction (in bold). (Abbreviations: ferritin light-chain, FTL; ferritin heavy-chain, FTH; heme oxygenase-1, HO-1; iron regulatory protein 2, IRP2; transferrin receptor, TfR; divalent metal transporter 1, DMT1; ceruloplasmin, Cp; ferroportin, Fpn; light-chain subunit of System X_c_^-^, xCT; 4-hydroxynonenal, 4-HNE; glutathione peroxidase 4, GPX4; NADPH oxidase 2, NOX2; ionized calcium-binding adaptor molecule 1, Iba1; glial fibrillary acidic protein, GFAP; triggering receptor expressed on myeloid cells 2, TREM2; endpoints, EP; branch length, BL; signal intensity, SI; arbitrary units, a.u.).

| **Cortex** | | | | | | |
| --- | --- | --- | --- | --- | --- | --- |
|  | **Saline Group** | **Iron Group** | **LPS Group** | **Iron+LPS Group** | **F(DFn,DFd)** | **p-value** |
| **Western blotting** | | | | | | |
| FTL | 0.21±0.07 | 0.25±0.04 | 0.31±0.05^a^ | 0.30±0.06^a^ | 3.956(3,24) | **0.0200** |
| FTH | 0.66±0.36 | 0.52±0.24 | 0.72±0.33 | 0.70±0.41 | 0.504(3,26) | 0.6830 |
| HO-1 | 1.95±0.33 | 2.20±0.35 | 2.16±0.53 | 2.16±0.67 | 0.372(3,24) | 0.7738 |
| IRP2 | 0.61±0.25 | 0.56±0.23 | 0.69±0.23 | 0.97±0.19^a,b^ | 4.225(3,25) | **0.0151** |
| TfR | 1.09±0.58 | 1.09±0.41 | 1.17±0.57 | 1.35±0.69 | 0.300(3,21) | 0.8253 |
| DMT1 | 1.36±0.28 | 1.37±0.24 | 1.18±0.44 | 1.70±0.34^c^ | 3.055(3,24) | **0.0478** |
| Cp | 0.81±0.43 | 0.75±0.36 | 0.76±0.17 | 0.65±0.19 | 0.365(3,25) | 0.7786 |
| Fpn | 0.67±0.26 | 0.64±0.15 | 0.66±0.20 | 0.67±0.15 | 0.049(3,25) | 0.9850 |
| xCT | 1.58±0.13 | 1.69±0.19 | 1.98±0.29^a^ | 2.01±0.38^a^ | 4.385(3,23) | **0.0140** |
| 4-HNE | 4.19±0.47 | 5.00±0.78 | 4.57±0.79 | 3.72±0.83^b^ | 3.424(3,21) | **0.0359** |
| GPX4 | 0.33±0.06 | 0.30±0.03 | 0.31±0.08 | 0.36±0.11 | 0.855(3,22) | 0.4788 |
| NOX2 | 1.56±0.26 | 1.47±0.38 | 1.41±0.38 | 1.51±0.28 | 0.271(3,24) | 0.8455 |
| Iba1 | 2.36±0.55 | 2.57±1.04 | 2.40±0.76 | 2.60±0.64 | 0.159(3,23) | 0.9228 |
| GFAP | 3.71±0.33 | 2.99±1.23 | 4.14±0.94 | 3.91±1.04 | 2.022(3,25) | 0.1365 |
| TREM2 | 2.02±0.65 | 1.95±0.74 | 2.38±0.61 | 2.16±0.47 | 0.660(3,24) | 0.5845 |
| **Immunohistochemistry** | | | | | | |
| Microglial EP/cell | 36.94  ±4.35 | 33.83  ±3.96 | 35.32  ±3.91 | 37.06  ±3.82 | 1.138(3,27) | 0.3515 |
| Microglial BL/cell | 275.47  ±28.14 | 253.26  ±58.91 | 300.14  ±65.34 | 383.34  ±112.92^a,b^ | 4.663(3,27) | **0.0094** |
| GFAP-SI (a.u.) | 34.81  ±3.64 | 32.92  ±4.27 | 35.44  ±3.25 | 38.83  ±3.71^b^ | 3.481(3,27) | **0.0295** |
| **Total reflection X-ray fluorescence** | | | | | | |
| Iron (mg/kg) | 69.57  ±18.17 | 71.75  ±12.68 | 69.46  ±7.08 | 63.81  ±5.80 | 0.540(3,23) | 0.6598 |
| Zinc (mg/kg) | 85.10  ±13.34 | 89.73  ±18.00 | 93.01  ±11.68 | 79.63  ±3.27 | 1.507(3,23) | 0.2392 |
| Copper (mg/kg) | 23.69  ±5.61 | 24.30  ±1.77 | 25.11  ±3.12 | 22.10  ±2.89 | 0.829(3,23) | 0.4914 |
| **Synchrotron radiation X-ray fluorescence** | | | | | | |
| Iron (mg/kg) | 82.17  ±19.86 | 76.00  ±13.22 | 73.18  ±12.01 | 83.21  ±23.85 | 0.575(3,27) | 0.6364 |
| Zinc (mg/kg) | 88.49  ±22.68 | 83.50  ±15.29 | 78.93  ±14.04 | 86.05  ±25.34 | 0.325(3,27) | 0.8076 |
| Copper (mg/kg) | 81.64  ±18.30 | 75.18  ±11.98 | 73.81  ±9.959 | 80.33  ±21.91 | 0.428(3,27) | 0.7347 |

Table S12: The effects of mild systemic iron and/or lipopolysaccharide (LPS) treatments on iron regulatory, ferroptosis and glia proteins; metals; and glia morphology in the striatum (Cohort 3: saline only [control group, n=7], iron only [n=8], LPS only [n=8] and iron+LPS group [n=8]). One-way ANOVA and *post hoc* Tukey correction to determine significant differences between saline, iron, LPS and iron+LPS groups. P ≤ 0.05 was considered significantly different from saline^a^, iron^b^ and LPS^c^, *post hoc* Tukey correction (in bold). (Abbreviations: ferritin light-chain, FTL; ferritin heavy-chain, FTH; heme oxygenase-1, HO-1; iron regulatory protein 2, IRP2; transferrin receptor, TfR; divalent metal transporter 1, DMT1; ceruloplasmin, Cp; ferroportin, Fpn; light-chain subunit of System X_c_^-^, xCT; acyl-CoA synthetase long chain fatty acid family member 4, ASCL4; 4-hydroxynonenal, 4-HNE; glutathione peroxidase 4, GPX4; NADPH oxidase 2, NOX2; ionized calcium-binding adaptor molecule 1, Iba1; glial fibrillary acidic protein, GFAP; triggering receptor expressed on myeloid cells 2, TREM2; endpoints, EP; branch length, BL; signal intensity, SI; arbitrary units, a.u.).

| **Striatum** | | | | | | |
| --- | --- | --- | --- | --- | --- | --- |
| **Proteins / metals** | **Saline Group** | **Iron Group** | **LPS Group** | **Iron+LPS Group** | **F(DFn,DFd)** | **p-value** |
| **Western blotting** | | | | | | |
| FTL | 1.07±0.32 | 0.89±0.27 | 0.79±0.08 | 1.01±0.35 | 1.607(3,27) | 0.2108 |
| FTH | 0.20±0.04 | 0.32±0.22 | 0.35±0.25 | 0.26±0.11 | 0.867(3,21) | 0.4740 |
| HO-1 | 0.47±0.35 | 0.42±0.29 | 0.49±0.22 | 0.51±0.26 | 0.133(3,26) | 0.9393 |
| IRP2 | 0.82±0.32 | 0.80±0.26 | 0.74±0.12 | 0.74±0.17 | 0.267(3,25) | 0.8487 |
| TfR | 0.55±0.31 | 0.92±0.59 | 1.41±0.91 | 1.00±0.71 | 1.868(3,25) | 0.1608 |
| DMT1 | 0.40±0.31 | 1.26±1.35 | 0.71±0.50 | 0.64±0.41 | 1.468(3,24) | 0.2483 |
| Cp | 0.64±0.09 | 0.62±0.10 | 0.79±0.28 | 0.83±0.28 | 1.855(3,25) | 0.1632 |
| Fpn | 0.85±0.32 | 0.60±0.14 | 0.54±0.12^a^ | 0.52±0.10^a^ | 4.089(3,23) | **0.0183** |
| xCT | 1.22±0.31 | 1.01±0.33 | 1.09±0.34 | 1.00±0.32 | 0.702(3,27) | 0.5591 |
| ACSL4 | 3.67±0.53 | 2.89±0.70 | 3.40±0.64 | 3.79±0.61^b^ | 3.085(3,26) | **0.0447** |
| 4-HNE | 4.08±2.20 | 3.76±2.57 | 3.18±2.35 | 2.81±2.10 | 0.461(3,27) | 0.7119 |
| GPX4 | 0.55±0.09 | 0.51±0.10 | 0.49±0.07 | 0.54±0.09 | 0.628(3,23) | 0.6042 |
| NOX2 | 0.26±0.06 | 0.25±0.05 | 0.24±0.03 | 0.29±0.06 | 1.001(3,23) | 0.4104 |
| Iba1 | 6.79±1.56 | 6.63±2.34 | 7.98±2.83 | 7.39±2.82 | 0.469(3,26) | 0.7067 |
| GFAP | 0.64±0.20 | 0.51±0.22 | 0.69±0.12 | 0.57±0.20 | 1.306(3,26) | 0.2935 |
| TREM2 | 1.85±0.44 | 0.77±0.34 | 1.39±0.89 | 1.09±0.46 | 1.347(3,24) | 0.2827 |
| **Immunohistochemistry** | | | | | | |
| Microglial EP/cell | 41.64  ±4.13 | 34.72  ±6.71^a^ | 36.48  ±3.14 | 39.02  ±3.21 | 3.311(3,27) | **0.0350** |
| Microglial BL/cell | 292.63  ±31.13 | 251.82  ±84.56 | 324.17  ±46.25 | 396.53  ±109.92^b^ | 5.162(3,27) | **0.0060** |
| GFAP SI (a.u.) | 31.95  ±3.47 | 30.14  ±6.90 | 35.83  ±3.38 | 34.15  ±5.29 | 1.902(3,26) | 0.1541 |
| **Total reflection X-ray fluorescence** | | | | | | |
| Iron (mg/kg) | 30.51  ±6.06 | 29.99  ±8.77 | 25.03  ±5.72 | 27.21  ±4.54 | 1.094(3,24) | 0.3706 |
| Zinc (mg/g) | 30.60  ±25.47 | 21.76  ±4.06 | 21.78  ±5.29 | 20.76  ±3.82 | 0.963(3,27) | 0.4244 |
| Copper (mg/kg) | 18.00  ±7.00 | 20.25  ±2.71 | 22.00  ±2.62 | 23.38  ±2.67 | 2.449(3,27) | 0.0853 |
| **Synchrotron radiation X-ray fluorescence** | | | | | | |
| Iron (mg/kg) | 41.86  ±10.55 | 44.79  ±8.17 | 40.46  ±7.54 | 42.95  ±9.31 | 0.336(3,27) | 0.7995 |
| Zinc (mg/kg) | 38.68  ±9.60 | 38.64  ±6.29 | 37.59  ±6.81 | 40.41  ±9.40 | 0.167(3,27) | 0.9178 |
| Copper (mg/kg) | 38.14  ±9.54 | 38.90  ±6.12 | 36.71  ±7.37 | 38.68  ±8.52 | 0.124(3,27) | 0.9452 |

Table S13: The effects of mild systemic iron and/or lipopolysaccharide (LPS) treatments on ionized calcium-binding adaptor molecule 1 (Iba1) and glial fibrillary acidic protein (GFAP) immunohistochemistry and metals in the substantia nigra (Cohort 3: saline only [control group, n=7], iron only [n=8], LPS only [n=8] and iron+LPS group [n=8]). One-way ANOVA and *post hoc* Tukey correction to determine significant differences between saline, iron, LPS and iron+LPS groups, p ≤ 0.05 was considered significantly different from saline^a^, iron^b^ and LPS^c^ post-Tukey correction (in bold). (Abbreviations: endpoints, EP; branch length, BL; signal intensity, SI; arbitrary units, a.u.).

| **Substantia nigra** | | | | | | |
| --- | --- | --- | --- | --- | --- | --- |
| **Proteins /metals** | **Saline Group** | **Iron Group** | **LPS Group** | **Iron+LPS Group** | **F(DFn,DFd)** | **p-value** |
| **Immunohistochemistry** | | | | | | |
| Microglial EP/cell | 36.87±6.77 | 33.38±8.04 | 35.52±6.22 | 38.20±3.45 | 0.757(3,25) | 0.5289 |
| Microglial BL/cell (μm) | 248.13  ±96.69 | 263.84  ±37.36 | 326.14  ±53.14 | 435.10  ±95.14^a,b,c^ | 9.164(3,25) | **0.0003** |
| GFAP SI | 37.17±2.59 | 35.76±4.28 | 38.30±2.92 | 38.60±2.06 | 1.230(3,24) | 0.3204 |
| **Synchrotron radiation X-ray fluorescence** | | | | | | |
| Iron (mg/kg) | 620.27  ±120.44 | 695.02  ±89.29 | 625.49  ±109.42 | 668.94  ±141.54 | 0.706(3,26) | 0.5574 |
| Zinc (mg/kg) | 519.49  ±143.06 | 603.61  ±113.00 | 535.52  ±112.84 | 565.93  ±135.58 | 0.645(3,26) | 0.5930 |
| Copper (mg/kg) | 475.62  ±93.97 | 513.15  ±67.85 | 460.73  ±74.41 | 481.90  ±78.46 | 0.596(3,26) | 0.6232 |
